# Encapsulation of curcumin in polymeric nanoparticles for antimicrobial Photodynamic Therapy

**DOI:** 10.1371/journal.pone.0187418

**Published:** 2017-11-06

**Authors:** Jeffersson Krishan Trigo Gutierrez, Gabriela Cristina Zanatta, Ana Laura Mira Ortega, Maria Isabella Cuba Balastegui, Paula Volpato Sanitá, Ana Cláudia Pavarina, Paula Aboud Barbugli, Ewerton Garcia de Oliveira Mima

**Affiliations:** Department of Dental Materials and Prosthodontics, School of Dentistry, Araraquara, São Paulo State University (UNESP), Araraquara, São Paulo, Brazil; Massachusetts General Hospital, UNITED STATES

## Abstract

Curcumin (CUR) has been used as photosensitizer in antimicrobial Photodynamic Therapy (aPDT). However its poor water solubility, instability, and scarce bioavalibility hinder its in vivo application. The aim of this study was to synthesize curcumin in polymeric nanoparticles (NP) and to evaluate their antimicrobial photodynamic effect and cytoxicity. CUR in anionic and cationic NP was synthesized using polylactic acid and dextran sulfate by the nanoprecipitation method. For cationic NP, cetyltrimethylammonium bromide was added. CUR-NP were characterized by physicochemical properties, photodegradation, encapsulation efficiency and release of curcumin from nanoparticles. CUR-NP was compared with free CUR in 10% dimethyl sulfoxide (DMSO) as a photosensitizer for aPDT against planktonic and biofilms (mono-, dual- and triple-species) cultures of *Streptococcus mutans*, *Candida albicans* and Methicillin-Resistant *Staphylococcus aureus*. The cytotoxicity effect of formulations was evaluated on keratinocytes. Data were analysed by parametric (ANOVA) and non-parametric (Kruskal-Wallis) tests (α = 0.05). CUR-NP showed alteration in the physicochemical properties along time, photodegradation similar to free curcumin, encapsulation efficiency up to 67%, and 96% of release after 48h. After aPDT planktonic cultures showed reductions from 0.78 log_10_ to complete eradication, while biofilms showed no antimicrobial effect or reductions up to 4.44 log_10_. Anionic CUR-NP showed reduced photoinactivation of biofilms. Cationic CUR-NP showed microbicidal effect even in absence of light. Anionic formulations showed no cytotoxic effect compared with free CUR and cationic CUR-NP and NP. The synthesized formulations improved the water solubility of CUR, showed higher antimicrobial photodynamic effect for planktonic cultures than for biofilms, and the encapsulation of CUR in anionic NP reduced the cytotoxicity of 10% DMSO used for free CUR.

## Introduction

Oral microbiota involves a wide range of species, including bacteria and fungi coexisting with each other in polymicrobial biofilms. Often, these microorganisms are inoffensive inhabitants of the oral cavity, but they can be infective pathogens and promote infections when an imbalance of the local or systemic homeostasis occurs. Among these species, *Streptococcus mutans* is the main etiological factor of dental caries due to its capacity to produce organic acids from dietary sugars (acidogenicity), especially sucrose, and to survive in low pH environment (aciduricity). Additionally, *S*. *mutans* may also be associated with severe systemic infections, such as acute endocarditis [1].

Other important microorganisms that should be highlighted are the fungi of the *Candida* genus, particularly *Candida albicans*, the most prevalent and virulent species [2]. This opportunistic pathogen is able to alter its morphology from yeast (blastopore) to filamentous forms (hyphal and pseudohyphal invasive forms), a process called polymorphism. The infection caused by *C*. *albicans*, called candidiasis, is one of the most common oral infections, especially in immunocompromised patients, affecting 90% of HIV-positive patients [3]. In more severe cases, the infection may spread to the blood stream and cause systemic infection, known as candidemia, whose mortality rate is high, varying between 40 and 60%, especially in patients in intensive care units [4].

Another pathogen that has been receiving great attention is Methicillin-Resistant *Staphylococcus aureus* (MRSA), responsible for high mortality rates in critically ill patients. MRSA is frequently found in long-stay hospital patients and an increasing trend in a community setting has also been reported [5]. Although MRSA is not considered part of the oral microbiota, it is able to co-exist with *C*. *albicans* in biofilms without antagonism and with bacteria cells preferentially associated to *C*. *albicans* hyphal invasive forms [6]. Similarly, *C*. *albicans* and *S*. *mutans* are also able to form mixed biofilms, beneficially affecting the growth of both microorganisms [7]. Thus, in addition to the fact that the three above-mentioned microorganisms are able to cause a relevant number of infections independently, they can also be commonly associated as co-infector microorganisms.

The widespread use of conventional antimicrobials (antibiotics and antifungals) has emerged the problem of microbial resistance [2,5], which results in persistence of infection, treatment failure, and side-effects of drugs especially when multiple antimicrobials are used. Due to these shortcomings of the currently available drug treatments, antimicrobial alternatives have been sought. A promising therapeutic modality for microbial inactivation is antimicrobial Photodynamic Therapy (aPDT), which uses the association of a photosensitizing agent (PS) [e.g., curcumin (CUR)] with a light at a suitable wavelength. The interaction between PS and light in the presence of oxygen results in the production of a reactive species, mainly the singlet oxygen and free radicals that promote cell damage and death [8,9].

Curcumin (diferuloymethane) is a polyphenol having a low molecular weight compound isolated from *Curcuma longa* L. It has been demonstrated that CUR presents antimicrobial effects [10] and recent studies have proved that the antifungal effect is potentiated when CUR is associated with blue light [11–13]. However, CUR is not water soluble, it has a limited bioavailability and instability, reasons that hinder its clinical applicability. An alternative in order to improve the properties of CUR is to encapsulate it in drug delivery systems, such as nanoparticles, nanoemulsions, and ciclodextrins [14]. Polylactic acid (PLA) is a natural, stable, biodegradable, and biocompatible polyester polymer used to encapsulate hydrophobic drugs [15].

Previous studies have encapsulated CUR in nanoformulations for antimicrobial purposes [16–20]. Hegge et al. [16] solubilized CUR in polyethylene glycol 400, Pluronic F127, and hydroxypropyl-γ-cyclodextrin and found the survival of *Staphylococcus epidermidis* biofilms to be between 13% and 29% after aPDT without significant differences between the solubilizing agents. CUR encapsulated in chitosan and polyethylene glycol had several effects in the absence of light: they inhibited the planktonic growth of MRSA and *Pseudomonas aeruginosa*, they caused a significant reduction in the number of both bacterial colonies, they reduced the amount of MRSA present in skin burns of mice, and they accelerated the healing process [17]. Using this same CUR encapsulation system at a concentration of 10 μg/mL, a complete elimination of the planktonic culture of the dermatophyte fungi *Trichophyton rubrum* was observed after the application of blue light at 10 J/cm^2^ [18]. The combination of silver nanoparticles (NP) with CUR in polymeric micelles inhibited the biofilm formation and reduced the biomass of *P*. *aeruginosa* and *S*. *aureus* [19]. Inhibition of biofilm formation was also observed when *S*. *mutans* was grown with CUR in chitosan, alginate, and starch [20]. Although these studies have evaluated the antimicrobial effect of CUR in nanoformulations, most investigations evaluated their antimicrobial effect in absence of light [17,19,20]. Moreover, few studies that evaluated the antimicrobial photodynamic effect of CUR are restricted to non-oral species [16,18].

Therefore, the purpose of the current study was to synthesize and characterize CUR-loaded into polymeric NP and to evaluate its cytotoxicity and photodynamic effects compared with free CUR as PSs against planktonic cultures and biofilms of oral species (*S*. *mutans* and *C*. *albicans*) and MRSA. The scientific hypothesis of this research was: water solubility and cytotoxity of CUR are improved with encapsulation in NP, whose antimicrobial effect is similar or higher than free CUR. Thus, the statistic hypothesis were: null hypothesis (H0)—CUR-NP does not improve water solubility, cytotoxicity and the antimicrobial photodynamic effect of CUR; alternative hypothesis (H1)—CUR-NP improves water solubity, cytotoxicity and the antimicrobial photodynamic effect of CUR. The synthesized formulations demonstrated solubility in water, and higher antimicrobial photodynamic effect for planktonic cultures compared with biofilms, and anionic CUR-NP showed no cytotoxicity compared with free CUR and cationic CUR-NP.

## Materials and methods

CUR (purity ≥ 65%), dextran sulfate (DEX), cetyltrimethylammonium bromide (CTAB) and bacitracin were purchased from Sigma Aldrich (St Louis, MO, USA). Dimethyl sulfoxide (DMSO), acetone PA/ACS, NaCl, sucrose were purchased from Labsynth *Produtos para Laboratório*, Ltda (Diadema, SP, Brazil). Brain Heart Infusion (BHI) was purchased from HiMedia Laboratories (Mumbai, India). Yeast Nitrogen Broth (YNB), Mitis Salivarius Agar (MSB), Mannitol Salt Agar (MSA) were purchased from Difco (Detroit, MI, USA). Tryptic Soy Broth (TSB), Sabouraud Dextrose Agar with 5 mg/mL of chloramphenicol (SDA) were purchased from Acumedia Manufactures Inc (Lansing, Michigan, USA).

### Photosensitizer and light source

Free CUR was used as a PS in ratio 9:1 of sterile distilled water: DMSO [11].

A Light Emitting Diodes (LED, LXHL- PR09, Luxeon^**®**^ III Emitter, Lumileds Lighting, San Jose, CA, USA) device having a wavelength from 440 nm to 460 nm (peak at 455 nm) and a light intensity of 33.58 mW/cm^2^ was designed by Physics Institute of São Carlos (University of São Paulo) and used as a light source for photodegradation and aPDT experiments.

### Encapsulation of CUR in polymeric nanoparticle (CUR-NP)

CUR-NP were synthesized using a nanoprecipitation method [21]. CUR stock solution at 4 mg/mL was prepared in acetone. For CUR encapsulation in NP, the aqueous phase containing DEX 1% in sterile ultrapure water was dropped into polymer/organic phase (PLA 0.5% in acetone) under moderate magnetic stirring. After 10 minutes of magnetic stirring, the solution was left resting at room temperature for 24 hours to completely evaporate the solvent (acetone). The resultant formulation had a negative (anionic) charge and maximum CUR concentration of 260 μM. PLA was kindly provided by Professor Valtencir Zucolotto from University of São Paulo.

To synthesize cationic (positive charge) CUR-NP, CTAB [22] 2% in sterile ultrapure water with NaCl 1.76% was added as surfactant after completely evaporating the solvent. Anionic and cationic NP without CUR were also synthesized as described above but without the CUR solution in the organic phase. This process was subjected to a patent deposited on National Institute for Industrial Property-INPI (process number BR 10 2016 015631 9).

### Characterization of CUR-NP

#### Physicochemical characterization

After encapsulation of CUR-NP, the samples were diluited in sterile ultrapure water (10 μL/mL) and characterized by Dynamic Light Scattering (DLS). The properties of average particle size, polydispersity index (PDI), and zeta potential were determined at 25°C in a Zetasizer Nano ZS (Malvern Instruments Ltd, Malvern, UK) in the absence of light. These properties of the formulations were evaluated for up to 40 days after the synthesis to determine the formulation stability.

#### Nanoparticles analysis by Field Emission Gun Scanning Electron Microscopy (FEG-SEM)

The CUR-NP diluted in sterile ultrapure water (1:50) were dropped on silicon conductive paper, which was placed in stub conductors. Samples were dried in a Sputter Coater apparatus (BAL-TEC SCD 050, Bridgend, UK) and observed in a FEG-SEM (JEOL model 7500F, Peabody, MA, USA) from the Institute of Chemistry, Araraquara, São Paulo State University (UNESP), Araraquara, SP, Brazil.

#### Absorption spectrum and photostability

Absorption spectrum of free CUR and both anionic and cationic CUR-NP at the concentration of 130 μM was determined in a UV-visible spectrophotometer (SQ 4802 UV-Visible Double Beam Spectrophotometer UNICO, United Products & Instruments Inc, Dayton, NJ, USA) at wavelengths range of 200–700 nm.

For the photodegradation test, free CUR and both anionic and cationic CUR-NP at 130 μM were illuminated by the LED device (see "Photosensitizer and light source" above) for four different times (5, 10, 15 and 20 minutes, corresponding to 10.8, 21.6, 32.4 and 43.2 J/cm^2^, respectively), and then the absorbances of the samples were read from the spectrophotometer as described above. The photostability of both CUR-NPs was compared with the free CUR.

#### Encapsulation efficiency (EE)

For the EE, samples of both CUR-NP at 52 μM were centrifuged at 6,000 x*g* for 10 minutes. The pellet (nanoparticles) was resuspended in ultrapure water and the absorbance of the samples was determined before (B) and after (A) centrifugation. Absorbance was determined in a UV-visible spectrophotometer at wavelengths that showed maximum absorbance for each sample [23]. The EE was calculated using the formula: EE% = A x 100/B.

#### Curcumin release from nanoparticles

The release test was carried out according to the technique proposed by Krausz et al. [17] with some modifications. Samples of both anionic and cationic CUR-NP were diluted in phosphate buffer saline (PBS- NaCl = 0.136 M; KH_2_PO_4_ = 1 mM; KCl = 2 mM; Na_2_HPO_4_ = 10 mM; pH 7.4) at a concentration of 52 μM and incubated at 37°C at 100 rpm. At interval of 2 hours, samples were centrifuged at 6,000 x*g* for 10 minutes to separate the nanoparticles from the solution, and then the nanoparticles (pellet) were diluted in acetone to solubilize the unreleased CUR, whose absorbance was determined by a UV-visible spectrophotometer. The total released CUR was calculated by dividing the absorbance read at each time interval by the absorbance of the encapsulated CUR estimated as maximum.

### Antimicrobial assays

#### aPDT against planktonic cultures of *S*. *mutans*, *C*. *albicans* and MRSA

Standard strains from the American Type Culture Collection (ATCC; Rockville, MD, USA) of *S*. *mutans* (UA159 ATCC 700610), *C*. *albicans* (ATCC 90028) and MRSA (ATCC 33591) were kept in -80°C in BHI with 1% of sucrose, YNB, and TSB, respectively, with 50% glycerol. Each strain was thawed and one loopful of each microorganism was spread on its respective agar plate (MSB supplemented with 15% sucrose and 0.2 IU/mL of bacitracin for *S*. *mutans*, SDA for *C*. *albicans*, and MSA for MRSA). The agar plates were then incubated at 37°C for 48 hours. All experiments with *S*. *mutans* were performed in a 5% CO_2_ incubator (L212 Laboven, Qingdao, China). After incubation, 5–10 colonies were resuspended in BHI for *S*. *mutans*, YNB for *C*. *albicans*, and TSB for MRSA, and the plates were incubated at 37°C for 16 hours. Aliquots of each suspension were diluted in each specific broth and incubated until optical density (OD) reached the mid-log phase of growth. The suspensions of each microorganism were standardized at OD of 0.613 arbitrary units (a.u.), 0.473 a.u. and 0.510 a.u. for *S*. *mutans*, *C*. *albicans* and MRSA, respectively, determinated in a spectrophotometer (Bioespectro SP 220 Equipar Ltda, Curitiba, PR, Brazil), equivalent to 10^7^−10^8^ CFU/mL for bacteria and 10^6^−10^7^ CFU/mL for yeast, respectively.

**Fig 1:**
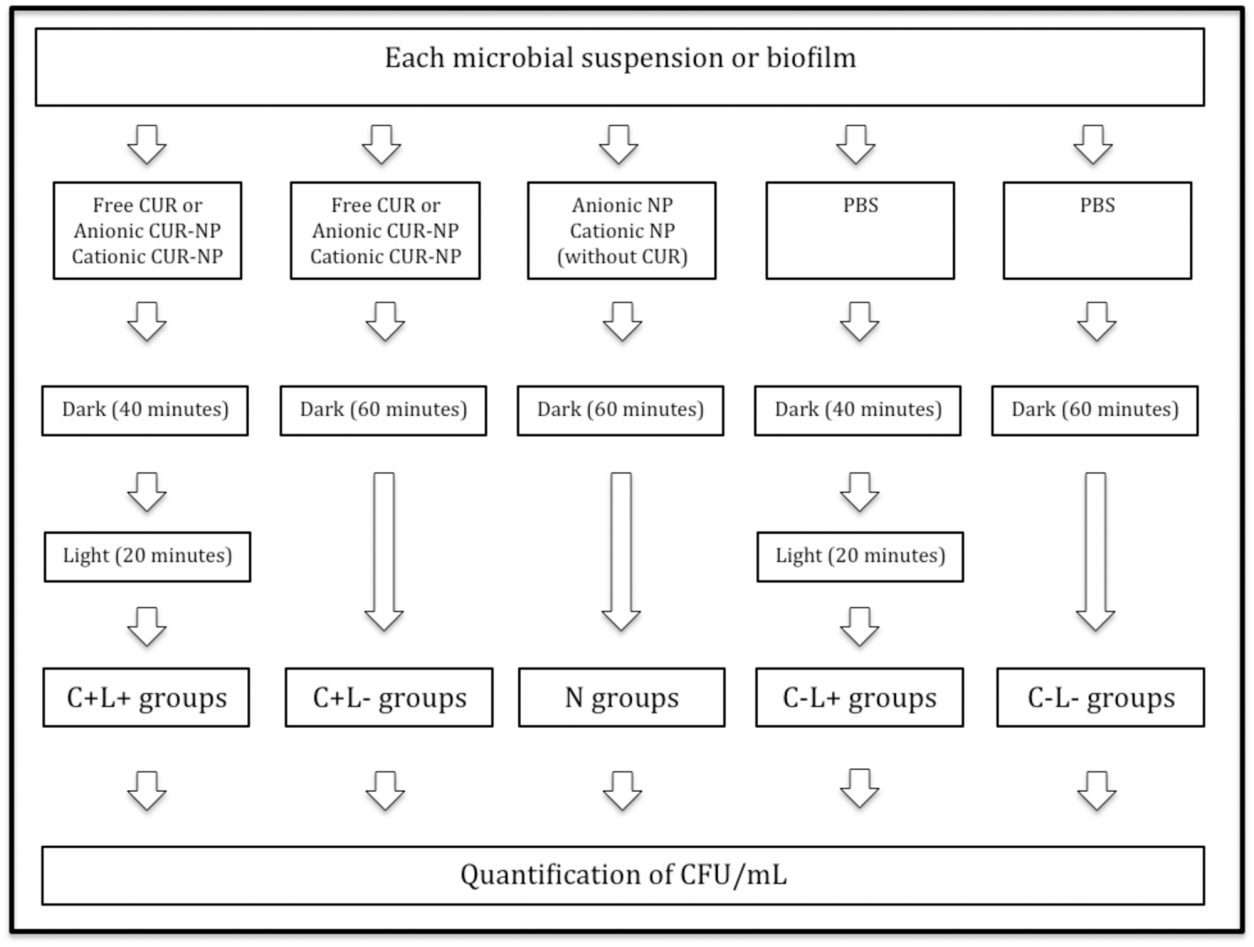
Diagram of the experimental conditions for planktonic cultures and biofilms.

Since the aim of this study was to evaluate the photoinactivation of biofilms, the aPDT parameters used for planktonic cultures was the same as those used for the biofilms in order to verify whether the aPDT efficacy was due to biofilm organization or to cell susceptibility to the synthesized formulations. These parameters were previously established in a pilot study (non-published data), in which CUR concentrations, the pre-irradiation times, and the illumination times were progressively increased from previous investigations [11–13] until photoinactivation of the multispecies biofilm was achieved. For aPDT, aliquots of 100 μL of each microbial suspension were individually transferred to 96-well flat-bottom microtiter plates (TPP Techno Plastic Products, Trasadingen, Switzerland). In each well, additional aliquots of 100 μL of the PS (free CUR or anionic or cationic CUR-NP at 260 μM) were added to each microbial suspension resulting in a final concentration of 130 μM. The plate was then kept in the dark for 40 minutes (pre-irradiation time) and, after this period, it was illuminated from above by LED light for 20 minutes, corresponding to a fluence of 43.2 J/cm^2^ (C+L+ groups). In order to evaluate the toxicity of the PS alone, samples of each microbial suspension were incubated with each PS (free CUR or anionic or cationic CUR-NP) and maintained in the dark for 60 minutes, corresponding to the pre-irradiation and illumination times (C+L- groups). The toxicity of the NPs were evaluated by incubating each microbial suspension with anionic or cationic NPs without CUR in the dark for 60 minutes (N groups). The effect of light alone was verified by adding 100 μL of PBS to each microbial suspension, incubating it for 40 minutes and illuminating it for 20 minutes (43.2 J/cm^2^, C-L+ group). The untreated control group did not receive any PS nor light (C-L-). Afterwards, each sample was submitted to tenfold serial dilutions that were spread on specific agar media for each microorganism as described previously. All agar plates were incubated at 37°C for 48 hours for colony quantification (CFU/mL). The diagram of all the experimental conditions is illustrated in Fig 1.

#### aPDT against mono-, dual- and triple-species biofilms

After the cultivation and standardization of each microorganism as described previously for the planktonic cultures, tests were made against mono-, dual-, and triple-species biofilms formed by *C*. *albicans*, *S*. *mutans*, and MRSA.

Biofilms were formed on a 96-well flat-bottom microtiter plate with a final microbial volume of 150 μL (150 μL of each microbial suspension for mono-species biofilms, 75 μL of each microbial suspension for dual-species biofilms, and 50 μL of each microbial suspension for triple-species biofilms). The plates were incubated at 37°C for 90 minutes (adhesion phase). Then, the culture broth was removed and wells were washed twice using PBS in order to remove the non-adherent cells. Afterwards, 150 μL of BHI were added to each well and the plate was incubated at 37°C for 48 hours to form the biofilms. After 24 hours, 75 μL of broth were removed from each well and the same volume of fresh BHI was added. All experiments with *S*. *mutans* were performed in a 5% CO_2_ incubator.

After the biofilm was formed, the medium broth was removed and the biofilm was washed twice with PBS. The same groups described for planktonic cultures were evaluated for the single and multispecies biofilm viability (C+L+, C+L-, N, C-L+, and C-L- / Fig 1). However, the concentration of free CUR was 1200 μM, since lower concentrations were not effective against these multispecies biofilms (non-published data), and the maximum concentration of the synthesized CUR-NP (260 μM for anionic and cationic formulations) was used. The same pre-irradiation time (40 minutes) and light fluence used for the planktonic cultures were employed for the biofilms. Additional samples (n = 3 or 6) of the triple-species biofilm had the PS (free CUR, anionic CUR-NP or cationic CUR-NP) washed thrice with PBS after pre-irradiation time and before illumination. After the treatments, the biofilms were mechanically disrupted using the pipette tip and tenfold serial dilutions were plated on specific agar medium for each microorganism as described previously. The agar plates were incubated at 37°C for 48 hours for colonies quantification (CFU/mL).

#### Confocal Scanning Laser Microscopy (CSLM) analysis

In order to evaluate the uptake of the synthesized formulations and the free CUR by the triple-species biofilm, samples were grown onto cover glass-bottom dishes (SPL Life Sciences, Pocheon, Korea) as described above in duplicate or triplicate. Biofilms were incubated for 40 minutes with free CUR, and anionic and cationic CUR-NP, and control biofilms were incubated for 40 minutes with SYTO-9 (Molecular Probes, Invitrogen Corp., Carlsbad, USA) prepared according to the manufacturer's instructions. For each group, samples were washed thrice with PBS or not before being observed under a confocal laser microscope (Carl Zeiss LSM 800 with Airyscan, Germany) with laser at 488 nm, and images were acquired using 40x oil-immersion objective and z-stack sections of 1 μm.

#### Cell viability assay

Spontaneously immortalized Normal Oral Keratinocytes (NOK-si) were kindly provided by Prof. Carlos Rossa Junior (Department of Periodontology, School of Dentistry, Araraquara, UNESP, Brazil) [24]. Cells were cultured in Dulbecco’s Modified Eagles Medium (DMEM-GIBCO BRL, Grand Island, NJ, USA) suplemeted with fetal bovine serum (10%), glutamine 2 mM (GIBCO BRL, Grand Island, NJ, USA) and penicillin 100 IU/mL-streptomycin 100 mg/mL (Sigma Chemical Co., St. Louis, MO, USA) [25]. Cells were grown at 37°C under 5% CO_2_ and 80% humidity (Series II water jacket CO_2_ incubator, Thermo Fisher Scientific, Marietta OH, USA) until 90% confluence.

A total of 20,000 cells quantified in a cell counter (Countess II FL, Lifetechnologies, Carlsbad, CA, USA) were transferred to wells of a black 96-well culture plate (NUNC; Thermo Scientific, Roskilde, Denmark). Cells were incubated for 40 minutes with the formulations (anionic CUR-NP, anionic NP, cationic CUR-NP and cationic NP), free CUR, 10% DMSO (free CUR vehicle), and Triton X-100 (positive control). For the negative control group, cells were grown with culture medium only. For all groups, DMEM was used as vehicle (anionic and cationic NP and CUR-NP were centrifuged at 6,000 x*g* for 10 minutes, and pellets were resuspended in DMEM). Then the cells were washed twice with PBS, and a solution of Alamar blue (Invitrogen,TREK Diagnostic Systems, OH, USA) and DMEM (1:9) was immediately added onto samples, which were incubated for 4 and 24 hours. After each period, the fluorescence intensity of the Alamar blue with its samples was mensured in a Fluoroskan Ascent FL fluorometer (Labsystems, Helsinki, Finland) having a 540 nm excitation wavelength and a 590 nm emission wavelength [26].

#### Statistical analyzes

The experiments were performed in triplicate on three different occasions for all experimental conditions unless otherwise stated. The CFU/mL values were transformed to logarithms. For the cytotoxic assay, the percentage of cell viability (survival) was calculated considering the negative control as 100%. Normal and homoscedastic data were analyzed by ANOVA and Tukey post-hoc tests. Normal and heteroscedastic data were analyzed by ANOVA/Welch and Games-Howell post-hoc tests. Non-normal data were evaluated by non-parametric Kruskall-Walis test. The significance level was 5%. Mean reductions in log_10_ were calculated only for normal data, since mean and standard deviation (SD) do not represent non-normal data. Therefore, normal data was plotted in bar charts as mean and SD, while non-normal data was plotted as box-plots.

## Results

### CUR-NP synthesis and characterization

#### Physicochemical characterization

With the nanoprecipitation method used, the maximum concentration of CUR encapsulated in the NPs was 260 μM. Higher concentrations resulted in CUR aggregation. Physicochemical properties of the nanoformulations are presented in Table 1. According to the DLS characterization, the mean values of the size, the PDI, and the zeta potential of the 4 formulations were similar at the baseline (day 0). As can be seen, these values have changed over time and, in general, the highest variations occurred after 15 days.

**Table 1:** DLS mean values of size, PDI and zeta potential (± standard deviation) of anionic CUR-NP, anionic NP, cationic CUR-NP and cationic NP.

Formulation	Days	Size (nm)	PDI (a.u.)	Zeta Potential (mV)
**Anionic CUR-NP**	0	237.9 (± 34.2)	0.08 (± 0.08)	-35.2 (± 6.52)
	7	259.1 (± 27.9)	0.22 (± 0.02)	-25.4 (± 2.95)
	15	270.2 (± 64.2)	0.16 (± 0.1)	-16.3 (± 2.64)
	35	273.3 (± 48.7)	0.27 (± 0.04)	-13.9 (± 5.17)
	40	326.1 (± 61.4)	0.28 (± 0.07)	-04.3 (± 3.32)
**Anionic NP**	0	203.6 (± 19.1)	0.10 (± 0.01)	-32.7 (± 0.96)
	7	266.9 (± 23.7)	0.21 (± 0.09)	-25.8 (± 1.76)
	15	284.3 (± 08.2)	0.25 (± 0.02)	-17.8 (± 2.24)
	35	289.4 (± 43.9)	0.29 (± 0.04)	-12.8 (± 5.44)
	40	399.5 (± 17.7)	0.31 (± 0.02)	-04.9 (± 0.96)
**Cationic CUR-NP**	0	239.0 (± 47.6)	0.20 (± 0.07)	+32.0 (± 1.45)
	7	305.6 (± 22.8)	0.27 (± 0.04)	+28.8 (± 5.75)
	15	256.6 (± 34.1)	0.29 (± 0.08)	+24.7 (± 1.89)
	35	266.9 (± 13.5)	0.25 (± 0.04)	+13.8 (± 0.53)
	40	279.9 (± 42.7)	0.25 (± 0.08)	+06.9 (± 3.23)
**Cationic NP**	0	205.3 (± 27.1)	0.20 (± 0.03)	+30.0 (± 1.36)
	7	248.8 (± 27.8)	0.24 (± 0.03)	+29.3 (± 2.51)
	15	297.6 (± 09.2)	0.32 (± 0.01)	+23.3 (± 3.90)
	35	263.3 (± 13.3)	0.25 (± 0.04)	+14.3 (± 1.48)
	40	290.3 (± 25.9)	0.30 (± 0.08)	+05.5 (± 1.62)

DLS: Dynamic Light Scattering; PDI: polydispersity index; a.u.: arbitrary units.

**Fig 2:**
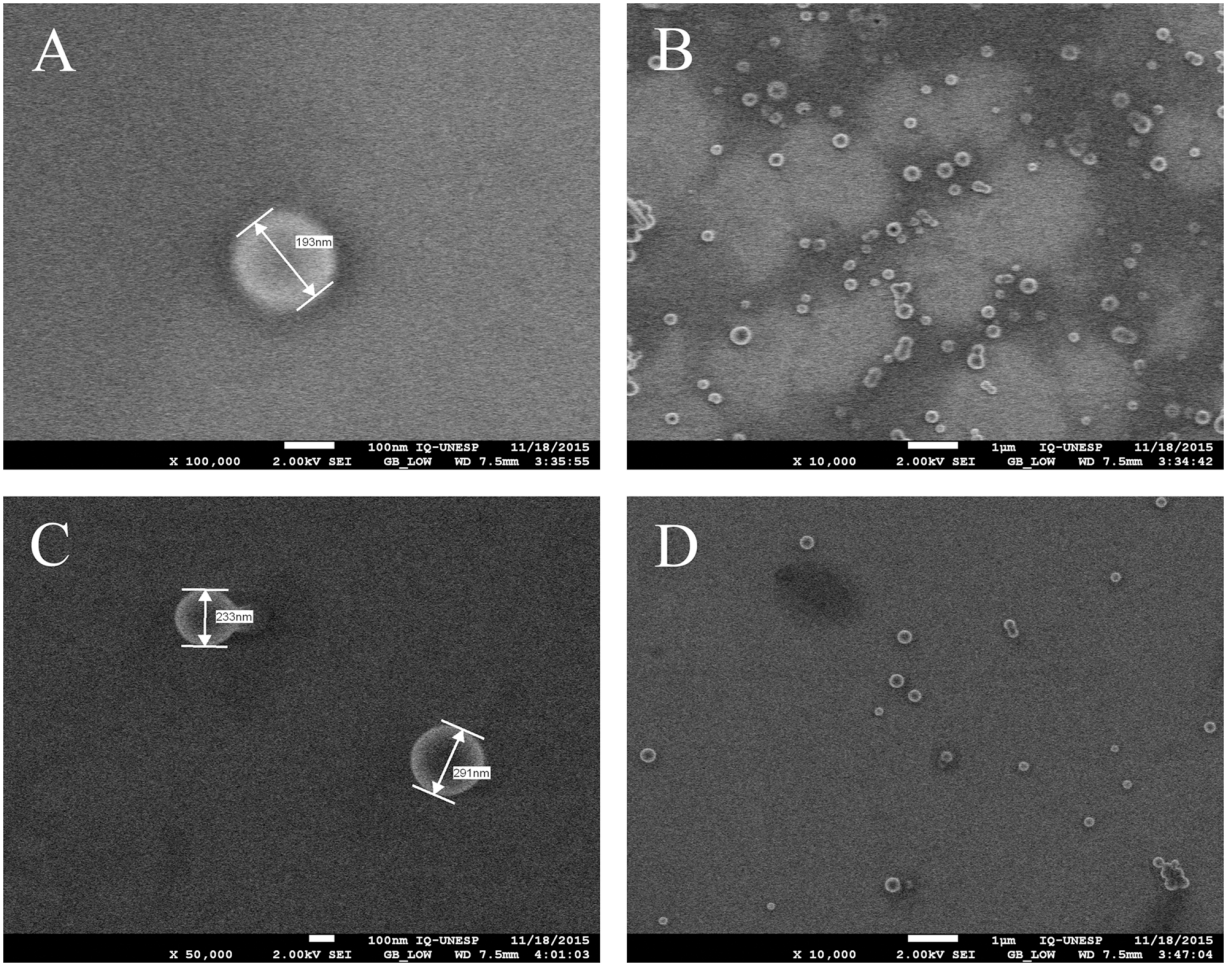
FEG-SEM of CUR–NP solutions. (A and B) Anionic CUR-NP solution; (C and D) cationic CUR-NP solution.

The FEG-SEM showed the CUR-NP having a spherical shape and sizes of 193 and 214 nm for the anionic and the cationic solutions, respectively, after their synthesis (Fig 2).

#### Absorption spectrum and photostability

**Fig 3:**
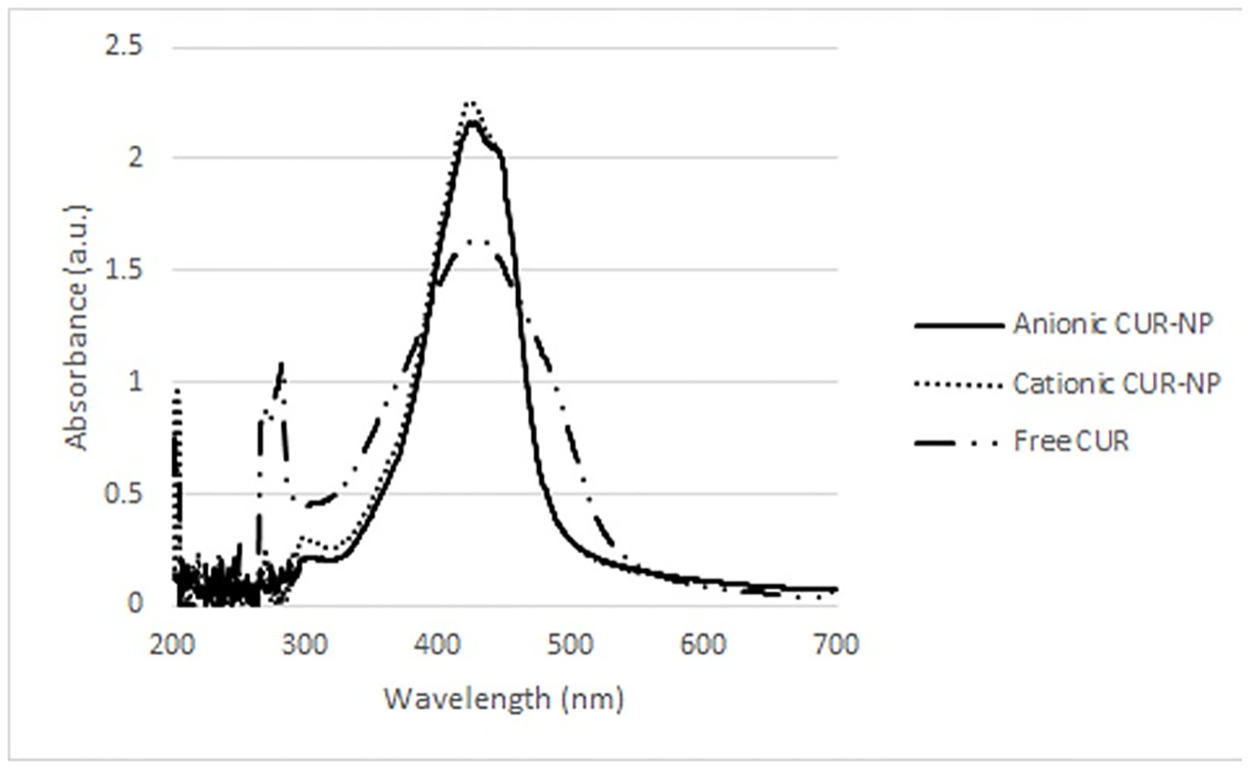
Absorption spectrum of free CUR, anionic CUR-NP, and cationic CUR-NP.

Fig 3 shows the absorption spectrum of the free CUR, and the anionic and cationic CUR-NPs. All PSs demonstrated maximum absorbance values from 1.645 a.u. to 2.272 a.u. at wavelengths of 425–429 nm (blue region).

**Fig 4:**
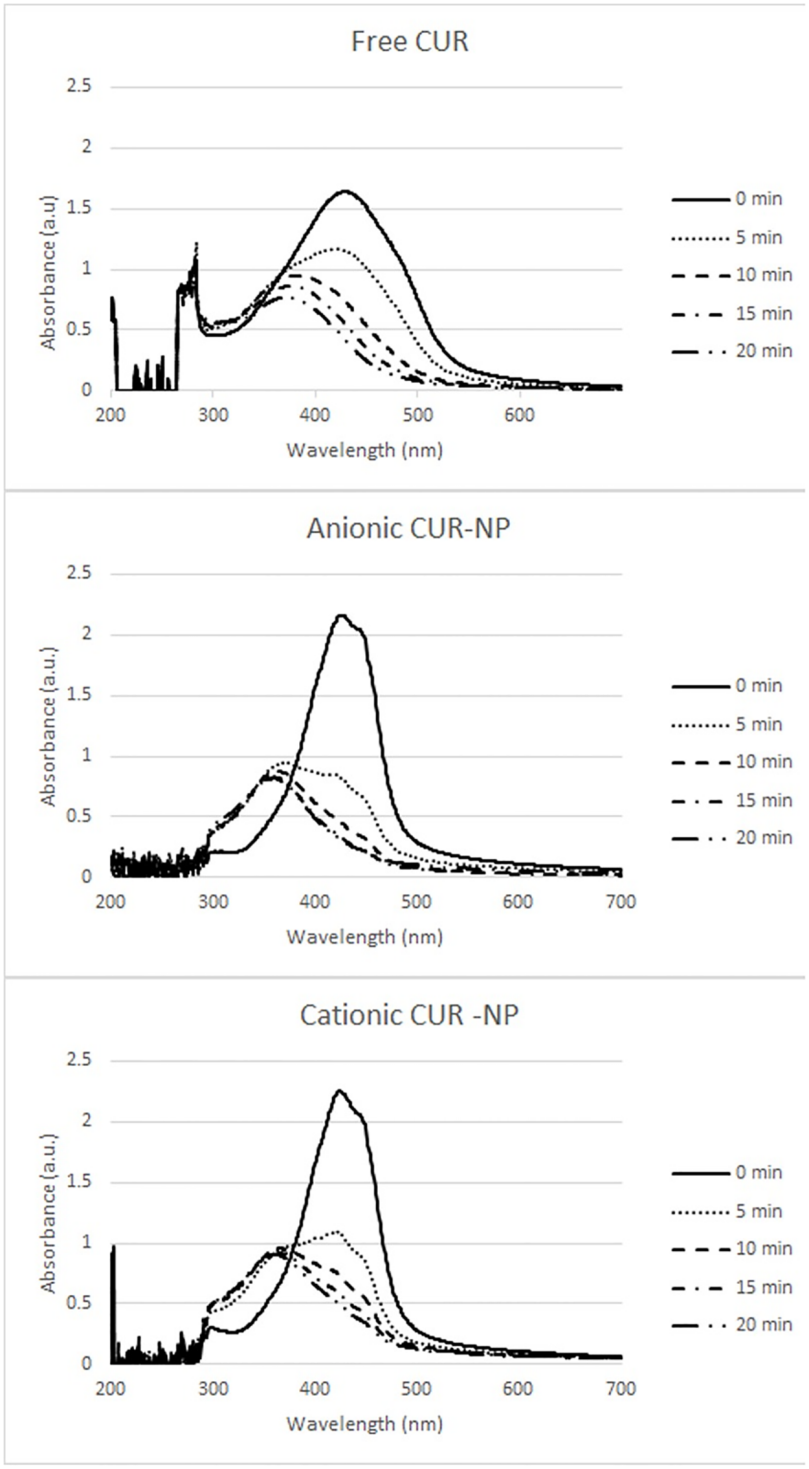
Photostability (values of absorbance) after different irradiation times observed for free CUR, anionic and cationic CUR-NP.

PS photostability is shown in Fig 4. The highest reduction of absorbance, verified after 43.2 J/cm^2^ of light (20 minutes), corresponded to 75.06%, 88.95%, and 71.67% of free CUR, anionic CUR-NPs and cationic CUR-NPs, respectively.

#### EE and CUR release

**Fig 5:**
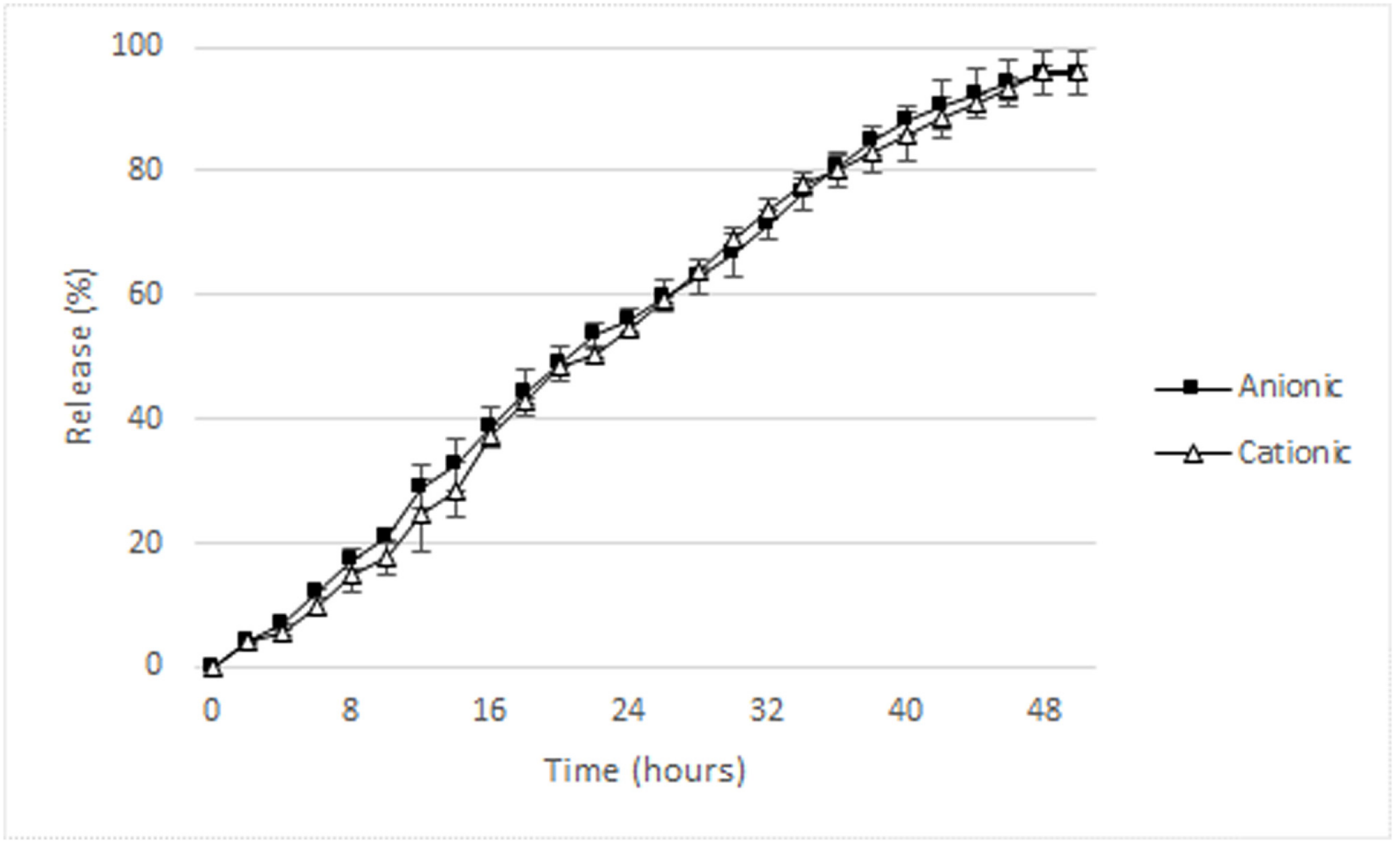
CUR release (% values) from anionic and cationic NP.

The mean EE of the anionic and cationic CUR-NP were 67% and 64.67%, respectively. Fig 5 shows CUR release from anionic and cationic NP. There was a gradual and incomplete CUR release over time and, after 48 hours, 96% of the CUR was released from both anionic and cationic NP.

### Antimicrobial assays

#### aPDT against planktonic cultures of *S*. *mutans*, *C*. *albicans* and MRSA

**Fig 6:**
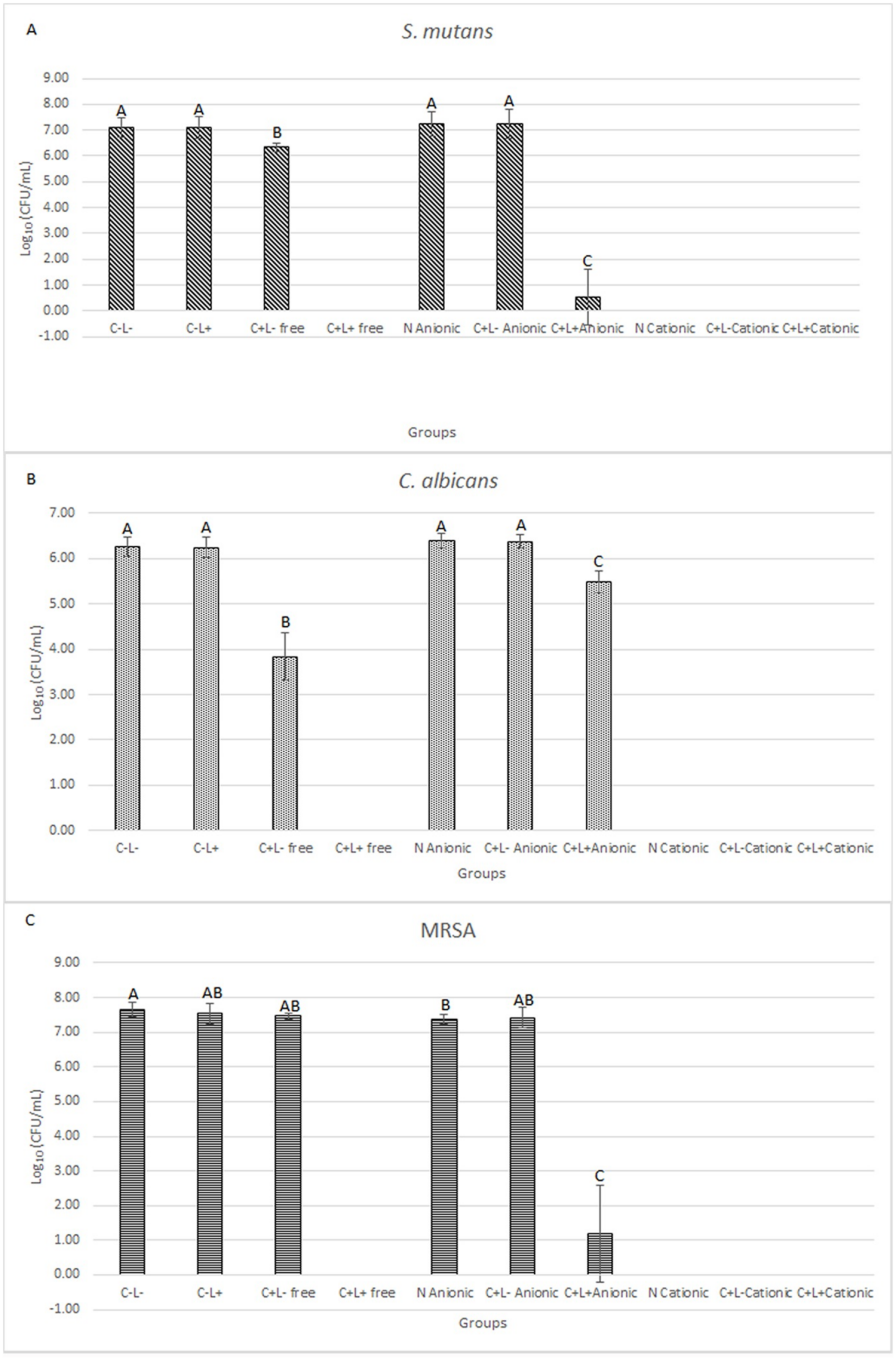
Mean values of log_10_(CFU/mL) of planktonic cultures. (A) *S*. *mutans*, (B) *C*. *albicans* and (C) MRSA. Error bars: standard deviation. The same letters show no statistical difference. C-L-: control group without PS nor light. C-L+: Planktonic culture treated with light only (43.2 J/cm^2^). C+L- free: Planktonic culture treated with free Cur only at 130 μM. C+L+ free: Planktonic culture submitted to aPDT with free Cur at 130 μM and 43.2 J/cm^2^. N Anionic: Planktonic culture treated with anionic NP without CUR. C+L- Anionic: Planktonic cultures treated only with anionic CUR-NP at 130 μM. C+L+ Anionic: Planktonic culture submitted to aPDT with anionic CUR-NP at 130 μM and 43.2 J/cm^2^. N Cationic: Planktonic culture treated with cationic NP without CUR. C+L- Cationic: Planktonic culture treated only with cationic CUR-NP at 130 μM. C+L+ Cationic: Planktonic culture submitted to aPDT with cationic CUR-NP at 130 μM and 43.2 J/cm^2^.

Fig 6 shows the results of planktonic cultures of *S*. *mutans* (Fig 6A), *C*. *albicans* (Fig 6B), and MRSA (Fig 6C).

The results obtained from planktonic cultures of *S*. *mutans* and *C*. *albicans* showed that treatments with aPDT mediated by free CUR (C+L+ free groups) resulted in the eradication of the microbial growth. In addition, the samples treated with free CUR only (C+L- groups with free CUR) showed a significant reduction of log_10_(CFU/mL) values (ANOVA/Welch; p<0.010 for bacteria and p<0.001 for yeast) when compared to the control group (C-L-; reduction of 0.75 log_10_ for *S*. *mutans* and 2.43 log_10_ for *C*. *albicans*). aPDT mediated by anionic CUR-NP (C+L+ anionic groups) also resulted in significant reductions (ANOVA/Welch; p<0.001) of the microbial growth compared with the other groups (C-L-, C-L+, N anionic, and C+L- anionic). Compared with the control, aPDT mediated by anionic CUR-NP showed reductions of 6.54 log_10_ and 0.78 log_10_ for *S*. *mutans* and *C*. *albicans*, respectively. It was also observed that cationic nanoparticles, with or without CUR (C+L- and N groups, respectively), eradicated the growth (no colony on agar plates) of *S*. *mutans* and *C*. *albicans*, indicating a microbicidal effect of cationic nanoparticles. Consequently, the samples treated with the cationic CUR-NP and light (C+L+ group) also showed no microbial growth.

For planktonic cultures of MRSA (Fig 6C), bacteria eradication was also observed for C+L+ free CUR, cationic N, cationic C+L-, and cationic C+L+ groups, similar to the outcomes observed for *S*. *mutans* and *C*. *albicans*. In addition, the samples treated with aPDT mediated by anionic CUR-NP (C+L+ group) resulted in significant reduction (p<0.001) of log_10_(CFU/mL) values compared with the other groups with bacterial growth (C-L-, C-L+, C+L- free, anionic N, and anionic C+L- groups). This reduction was equivalent to 6.47 log_10_ compared to the control group. In opposition to the results verified for *S*. *mutans* and *C*. *albicans*, MRSA samples treated with free CUR (C+L- group) showed no significant difference (p≥0.261) compared to the C-L-, C-L+, N, and C+L- (anionic formulation) groups. Additionally, MRSA samples treated with anionic NP without CUR (N group) showed a significant difference (p = 0.048) compared to the control. Despite the fact that this difference may be statistically significant, it was not relevant from the microbiological point of view, since the mean values of the control and the anionic N groups were 7.64 and 7.36 log_10_(CFU/mL), a difference lower than 1 log_10_ (0.28 log_10_).

#### aPDT against mono-species biofilms

**Fig 7:**
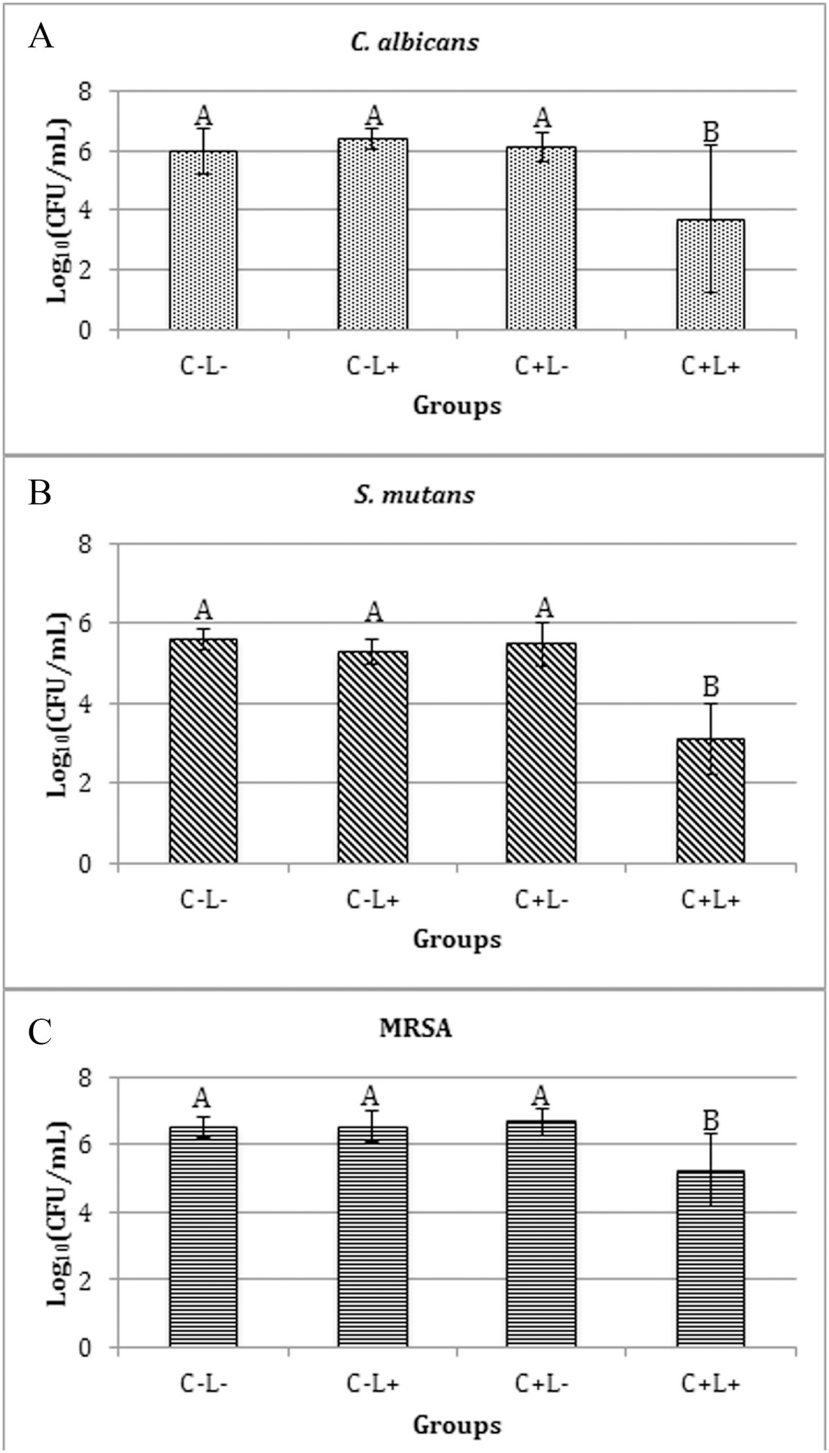
Values of log_10_(CFU/mL) of mono-species biofilm treated with free CUR. (A) *C*. *albicans*, (B) *S*. *mutans* and (C) MRSA. Bars charts show mean and standard deviation (error bars). The same letters show no statistical difference. C-L-: Control group without PS nor light. C-L+: Biofilm treated only with light (43.2 J/cm^2^). C+L-: Biofilm treated only with Cur at 1200 μM. C+L+: Biofilm submitted to aPDT with Cur at 1200 μM and 43.2 J/cm^2^.

When the free CUR was evaluated, a significant reduction of log_10_(CFU/mL) of biofilms of *C*. *albicans* (p≤0.043), *S*. *mutans* (p<0.001) and MRSA (p≤0.05) (Fig 7A, 7B and 7C) was observed after the aPDT compared with the control (C-L-), which did not show a significant difference (p>0.05) compared with the other groups (C+L- and C-L+). The mean reductions observed for each species were: 1.24, 2.26, and 2.47 log_10_ for MRSA, *C*. *albicans* and *S*. *mutans*, respectively.

**Fig 8:**
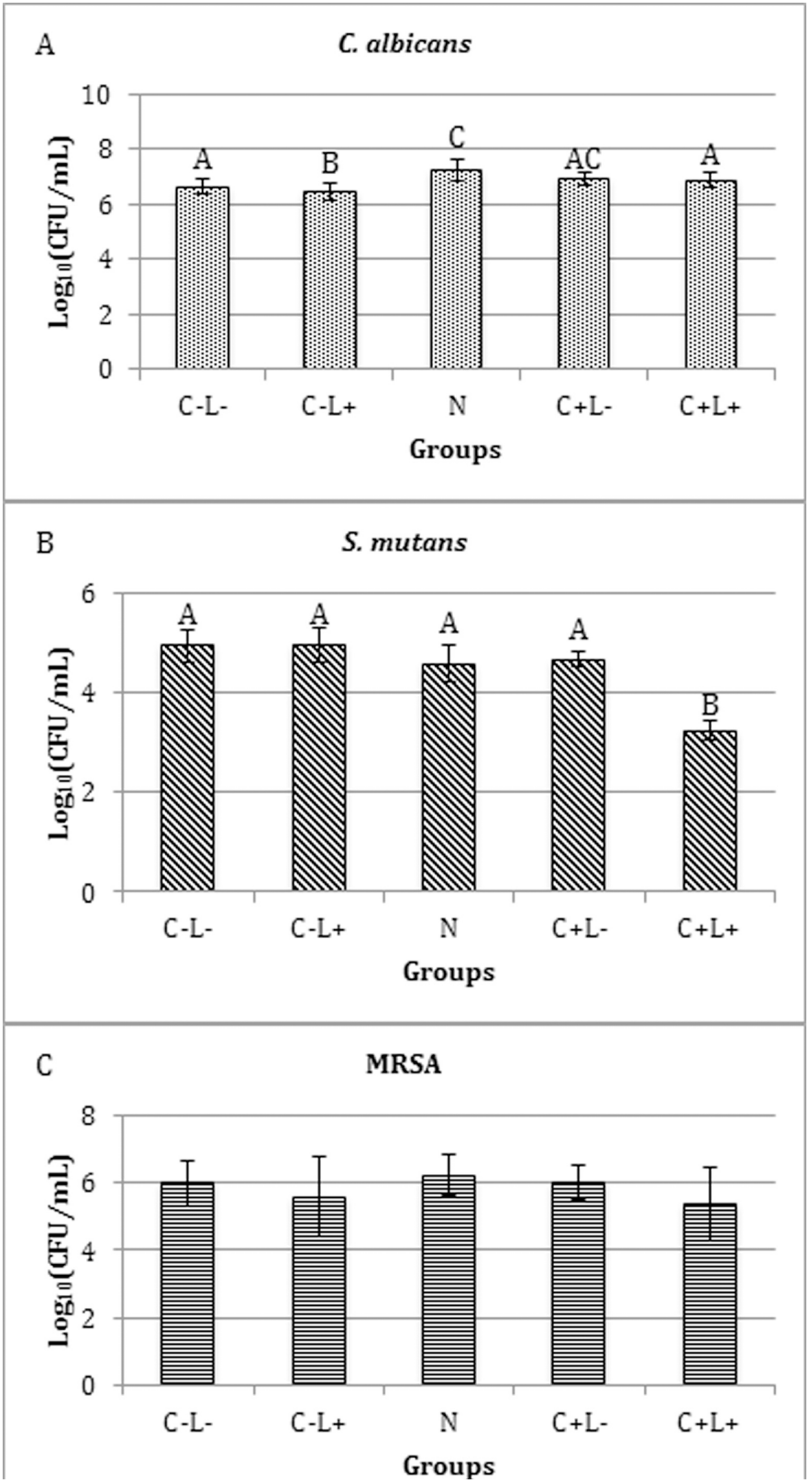
Values of log_10_(CFU/mL) of mono-species biofilm treated with anionic CUR-NP. (A) *C*. *albicans*, (B) *S*. *mutans* and (C) MRSA. Bars charts show mean and standard deviation (error bars). The same letters show no statistical difference. C-L-: Control group without PS nor light. N: Biofilm treated with anionic NP without CUR. C-L+: Biofilm treated only with light (43.2 J/cm^2^). C+L-: Biofilm treated only with anionic CUR-NP at 260 μM. C+L+: Biofilm submitted to aPDT with anionic CUR-NP at 260 μM and 43.2 J/cm^2^.

For the anionic CUR-NPs, aPDT promoted no significant difference (p≥0.532) in the viability of *C*. *albicans* biofilm compared with the control (C-L-) and C+L- groups. However a significant difference (p≤0.038) was observed between the N group and the C-L-, C-L+ and C+L+ groups, and also between the C-L+ group and the C+L- and C+L+ groups (p≤0.034) (Fig 8A). For *S*. *mutans* (Fig 8B), the aPDT mediated by the anionic CUR-NP promoted a significant reduction (p<0.001) in its viability compared with the other groups, which showed no significant difference (p≥0.067) among them. Compared with the control (C-L-) group, a reduction of 1.70 log_10_ was verified. Conversely, ANOVA demonstrated no significant difference (p = 0.222) for MRSA (Fig 8C).

**Fig 9:**
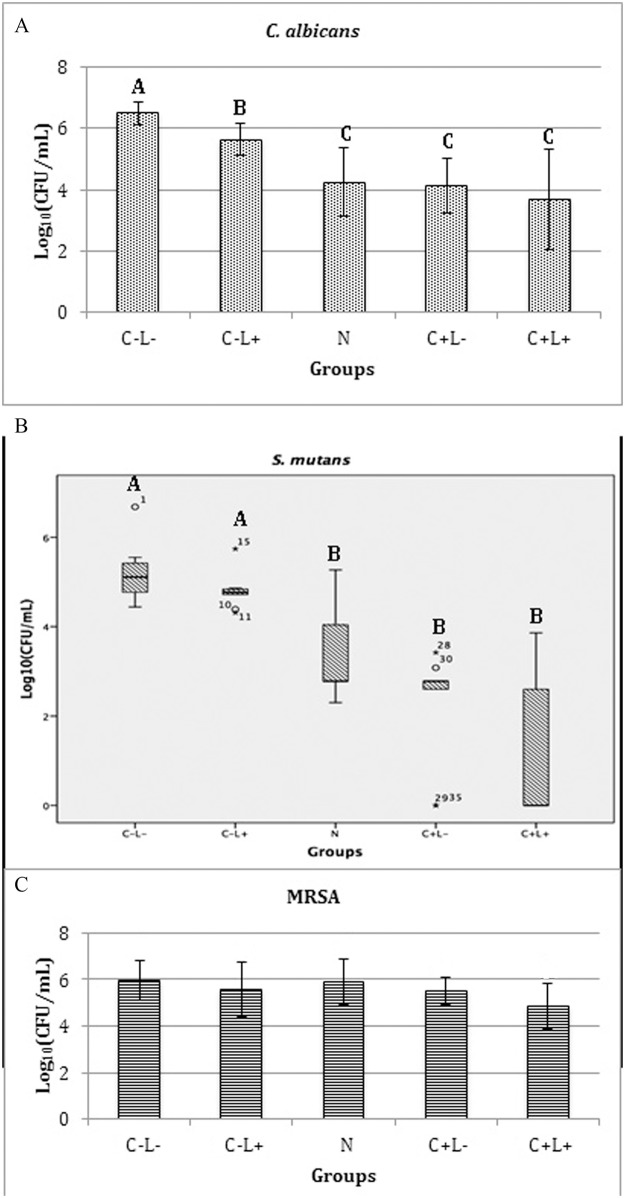
Values of log_10_(CFU/mL) of mono-species biofilms treated with cationic CUR-NP. (A) *C*. *albicans*, (C) MRSA. Bars charts show mean and standard deviation (error bars). (B) Box-plot of *S*. *mutans* shows median (dash) first and third quartiles (boxes), highest and lowest values (error bars). Bar charts were used for normal data submitted to parametric analysis, while box-plot was used for non-normal data submitted to non-parametric analysis. The same letters show no statistical difference. C-L-: Control group without PS nor light. N: Biofilm treated with cationic NP without CUR. C-L+: Biofilm treated only with light (43.2 J/cm^2^). C+L-: Biofilm treated only with cationic CUR-NP at 260 μM. C+L+: Biofilm submitted to aPDT with cationic CUR-NP at 260 μM and 43.2 J/cm^2^.

The evaluation of the cationic CUR-NPs against *C*. *albicans* demonstrated that the values of all experimental groups (C+L-, C-L+, N and C+L+) were significantly lower (p≤0.008) than the control group (C-L-). The fungal monospecies biofilm submitted to light only showed log_10_(CFU/mL) values significantly (p≤0.041) lower than the control and higher than the other groups. The groups treated with cationic CUR-NPs with and without light, and with NPs without CUR (C+L-, C+L+ and N) showed no significant difference among themselves. When *S*. *mutans* was evaluated, the non-parametric Kruskal-Wallis test demonstrated that biofilm treated with cationic NPs (N, C+L- and C+L+ groups) were significantly lower (p≤0.010) than the control and light-only groups (C-L- and C-L+). For biofilms of MRSA, ANOVA demonstrated no significant difference (p = 0.108) (Fig 9A, 9B and 9C).

#### aPDT against dual-species biofilms

**Fig 10:**
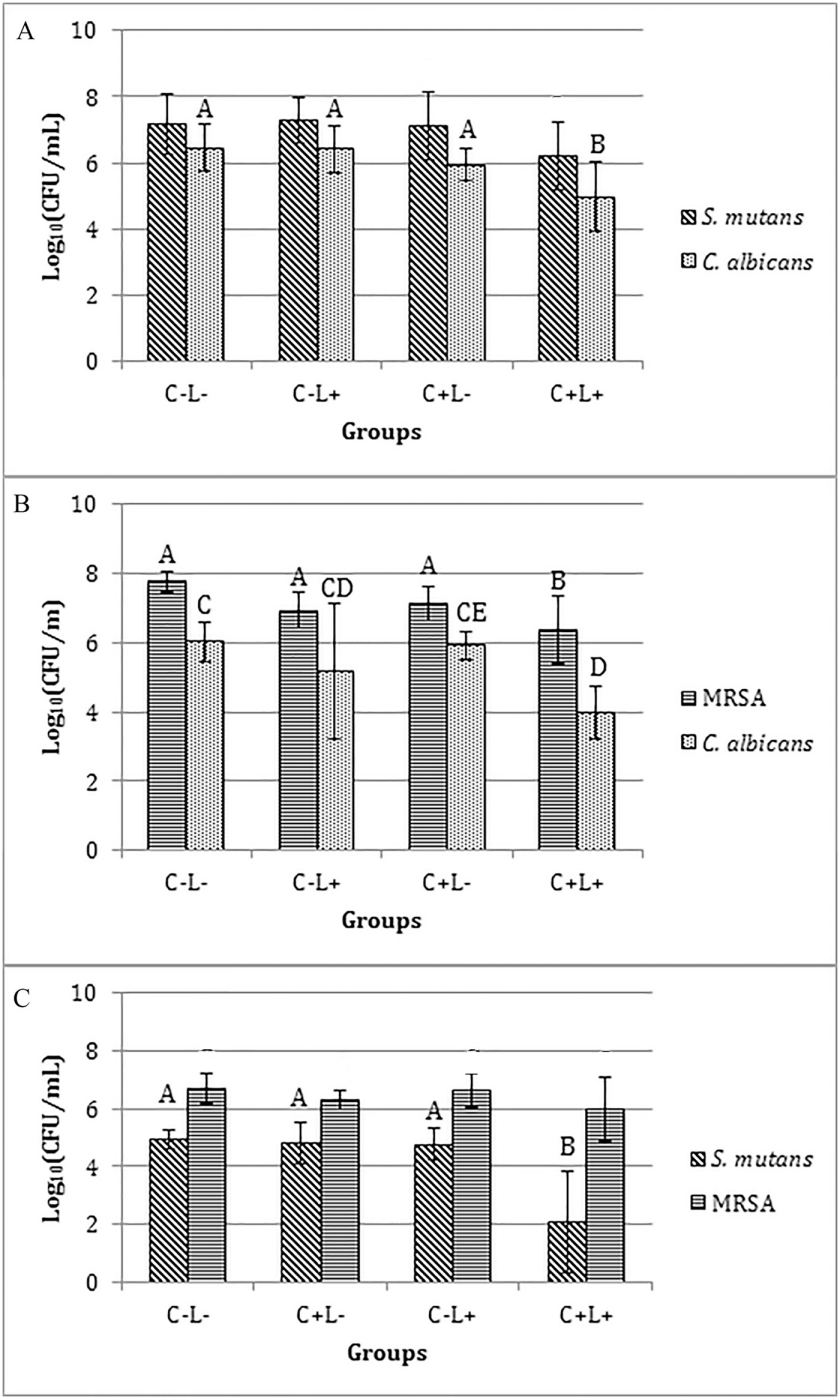
Values of log_10_(CFU/mL) of dual-species biofilm treated with free CUR. (A) *S*. *mutans* and *C*. *albicans*, (B) MRSA and *C*. *albicans*, (C) *S*. *mutans* and MRSA. Bars charts show mean and standard deviation (error bars). The same letters show no statistical difference. C-L-: Control group without PS nor light. C-L+: Biofilm treated only with light (43.2 J/cm^2^). C+L-: Biofilm treated only with free Cur at 1200 μM. C+L+: Biofilm submitted to aPDT with free Cur at 1200 μM and 43.2 J/cm^2^.

For the free CUR, the biofilm of *S*. *mutans* and *C*. *albicans* (Fig 10A) showed a significant reduction (p≤0.002) of 1.50 log_10_ after the aPDT only for *C*. *albicans* compared with the control (C-L-) and C-L+ groups, which did not differ significantly (p>0.05) from the C+L- group. On the other hand, *S*. *mutans* showed no significant difference (p>0.05) among all the groups evaluated. On the other hand, the biofilms of MRSA and *C*. *albicans* (Fig 10B) showed a significant reduction in the viability of both species after aPDT equivalent to 1.39 log_10_ (p<0.001) and 2.06 log_10_ (p = 0.002), respectively. For *C*. *albicans*, a significant difference (p = 0.004) was also observed between the C+L+ and C+L- groups and no significant difference (p≥0.130) among C-L-, C-L+ and C+L- groups and similarly none between C+L+ and C-L+ groups. For the biofilms of *S*. *mutans* and MRSA (Fig 10C), the ANOVA/Welch demonstrated a significant effect (p = 0.003) in the viability of *S*. *mutans*, since a significant (p = 0.005) reduction of 2.86 log_10_ after aPDT was observed compared with the control group. aPDT also promoted a significant reduction (p≤0.007) compared with the C-L+ and C+L- groups, which did not differ significantly (p≥0.813) from the control. On the other hand, ANOVA/Welch did not demonstrate a significant (p = 0.156) difference in the viability of MRSA among the groups.

**Fig 11:**
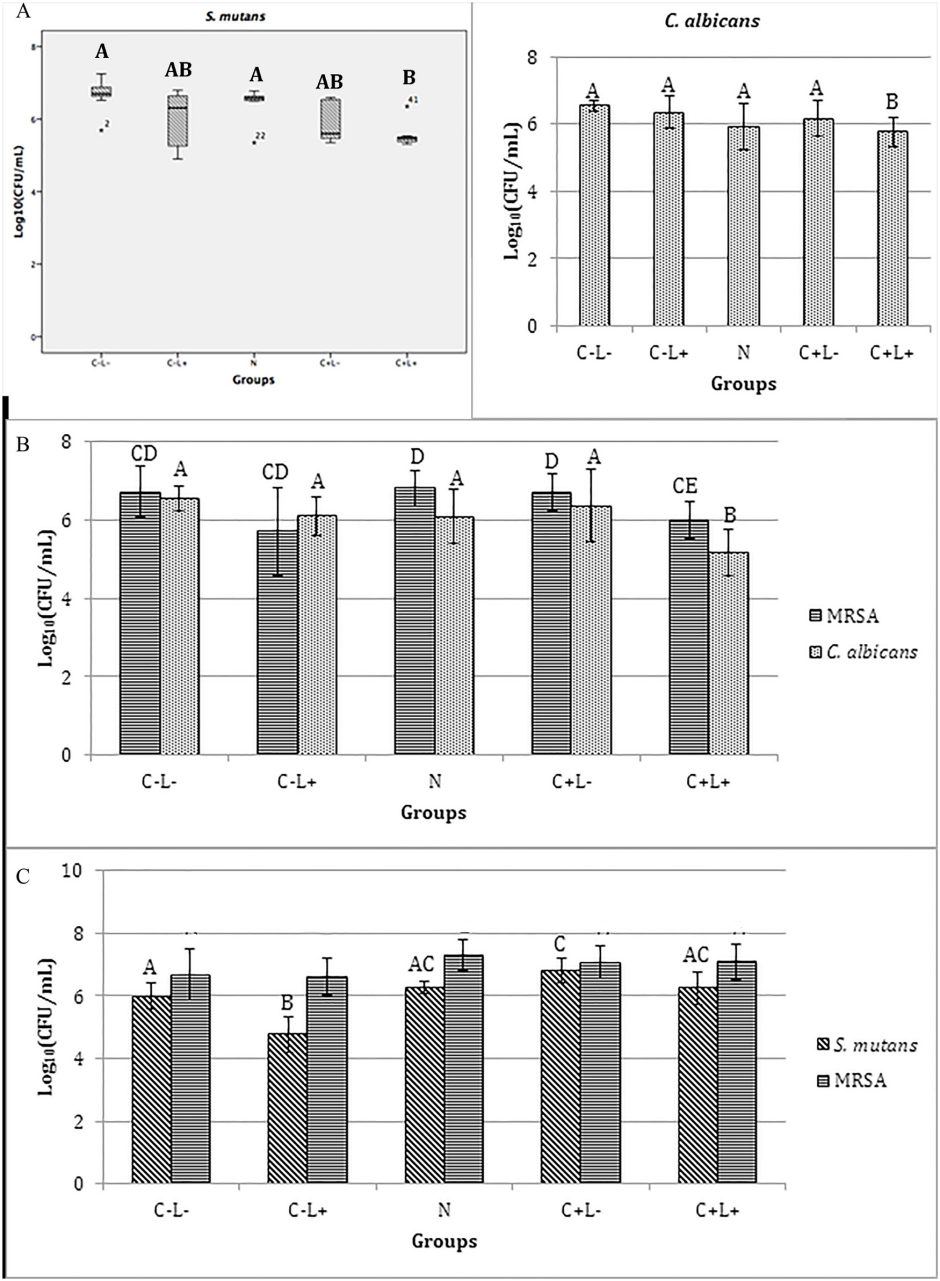
Values of log_10_(CFU/mL) of dual-species biofilms treated with anionic formulation. (A) *S*. *mutans* and *C*. *albicans*, (B) MRSA and *C*. *albicans*, and (C) *S*. *mutans* and MRSA. Bars charts show mean and standard deviation (error bars) values of log_10_(CFU/mL). Box-plot shows median (dash), first and third quartiles (boxes), highest and lowest values (error bars). The same letters showed no statistical difference. Bar charts were used for normal data submitted to parametric analysis, while box-plot was used for non-normal data submitted to non-parametric analysis. C-L-: Control group without PS nor light. N: Biofilm treated with anionic NP without CUR. C-L+: Biofilm treated only with light (43.2 J/cm^2^). C+L-: Biofilm treated only with anionic CUR-NP at 260 μM. C+L+: Biofilm submitted to aPDT with anionic CUR-NP at 260 μM and 43.2 J/cm^2^.

The evaluation of the anionic CUR-NPs against the biofilm of *S*. *mutans* and *C*. *albicans* demonstrated, for *S*. *mutans* according to the Kruskal-Wallis test, a significant difference (p = 0.010) between the C+L+ group (aPDT) and the N and C-L- (control) groups. The other groups showed no significant difference (p≥0.184) among themselves. For *C*. *albicans* viability, the aPDT resulted in a significant reduction (p = 0.004) of 0.78 log_10_ compared with the control (C-L-) group. The other groups showed no significant difference (p≥0.106) among themselves (Fig 11A).

In the biofilm of MRSA and *C*. *albicans*, the aPDT mediated by the anionic CUR-NP resulted in a significant difference (p≤0.033) in the fungal viability compared with the other groups, which showed no significant difference (p≥0.536) among themselves. Compared with the control group (C-L-), the aPDT reduced the viability of *C*. *albicans* by 1.37 log_10_. On the other hand, for MRSA, no significant reduction (p≥0.950) was verified between the C+L+ group (aPDT) and the control (C-L-) and the C-L+ groups. Significant differences (p≤0.033) were observed only between the C+L+ group (aPDT) and the N and C+L- groups (Fig 11B).

For the biofilm of *S*. *mutans* and MRSA, the aPDT using the anionic CUR-NP promoted no significant difference (p≥0.063) in the viability of *S*. *mutans* compared with the other groups, except for the C-L+ group, which showed a significant difference (p<0.001) compared with the other groups. A significant difference (p = 0.003) was also observed between the C+L- groups and the control (C-L-) groups. For MRSA, ANOVA demonstrated no significant difference (p = 0.094) among the groups (Fig 11C).

**Fig 12:**
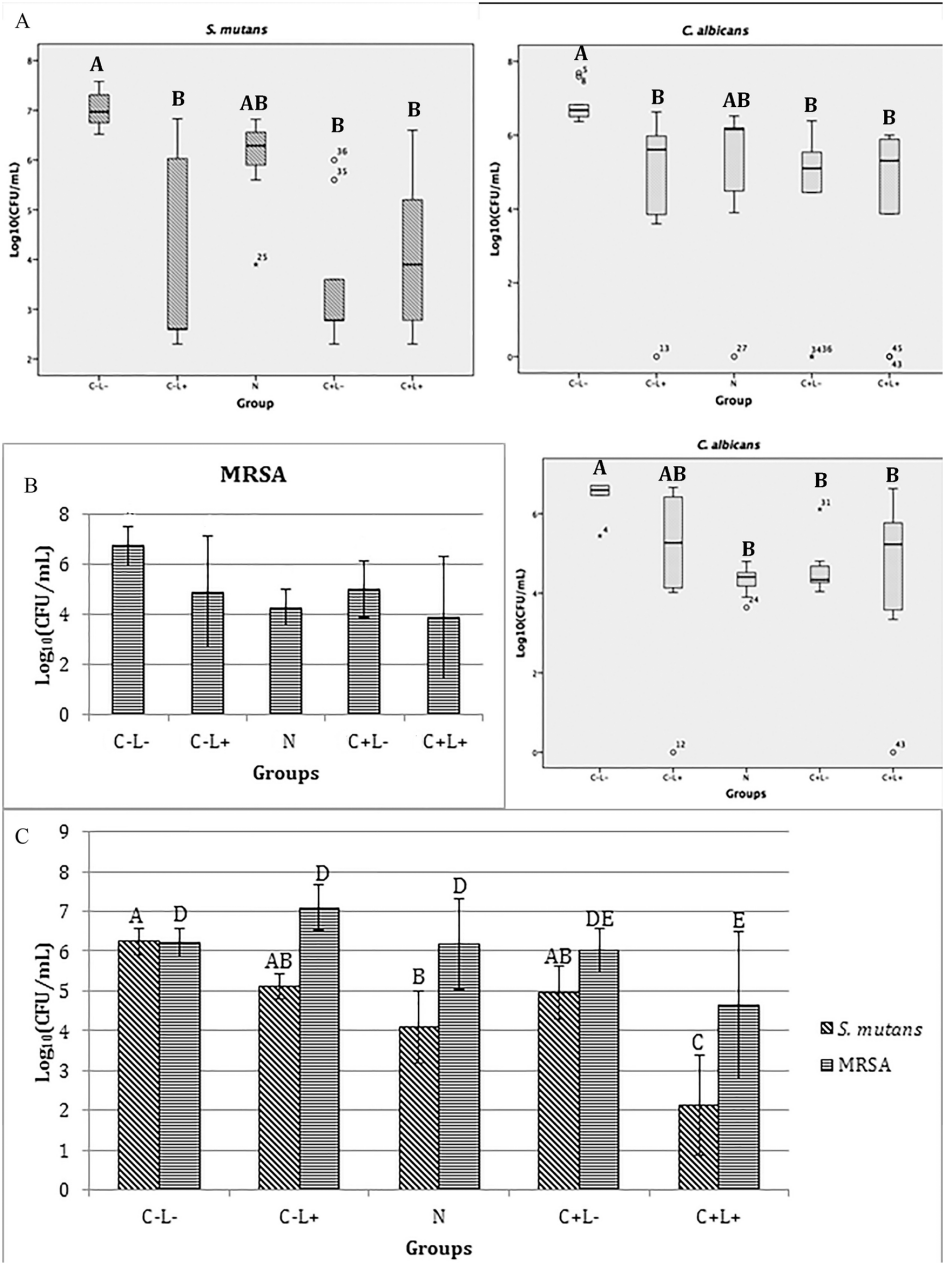
Values of log_10_(CFU/mL) of dual-species biofilms treated with cationic formulation. (A) *S*. *mutans* and *C*. *albicans*, (B) MRSA and *C*. *albicans*, and (C) *S*. *mutans* and MRSA treated with cationic CUR-NP. Bars charts show mean and standard deviation (error bars) values of log_10_(CFU/mL). Box-plot shows median (dash), first and third quartiles (boxes), highest and lowest values (error bars). The same letters showed no statistical difference. Bar charts were used for normal data submitted to parametric analysis, while box-plot was used for non-normal data submitted to non-parametric analysis. C-L-: Control group without PS nor light. N: Biofilm treated with cationic NP without CUR. C-L+: Biofilm treated only with light (43.2 J/cm^2^). C+L-: Biofilm treated only with cationic CUR-NP at 260 μM. C+L+: Biofilm submitted to aPDT with cationic CUR-NP at 260 μM and 43.2 J/cm^2^.

When the cationic CUR-NPs were evaluated against the biofilm of *S*. *mutans* and *C*. *albicans*, the Kruskal-Wallis test demonstrated a significant difference in the viability of both species (p≤0.004 and p≤0.005, respectively) between the control group (C-L-) and the other groups (C+L+, C+L- and C-L+), except for the N group (p = 0.708 and p = 0.089, respectively). The other groups did not show a significant difference (p≥0.134 and p = 1.000) (Fig 12A).

In the biofilm of MRSA and *C*. *albicans*, ANOVA demonstrated no significant (p = 0.206) difference for MRSA. Conversely for *C*. *albicans*, the Kruskal-Wallis test showed a significant (p≤0.010) difference between the control group (C-L-) and the N, C+L- and C+L+ groups, which showed no significant difference among themselves (p = 1.000). The C-L+ group did not show any significant difference (p = 1.000) either when compared with the other groups (Fig 12B).

Finally, in the biofilm of *S*. *mutans* and MRSA, the aPDT using the cationic CUR-NPs promoted a significant (p<0.001) reduction in the viability of *S*. *mutans* compared with the other groups, with a reduction of 4.11 log_10_ compared with the control (C-L-). A significant difference (p = 0.009) was also observed between the N group and the control (C-L-), while the other groups showed no significant difference (p≥0.208) among themselves. For MRSA, the aPDT promoted a significant (p≤0.029) reduction in its viability compared with the other groups, except for the C+L- group (p = 0.058). The reduction in the aPDT group (C+L+) was equivalent to 1.58 log_10_ compared with the control group (C-L-). The other groups demonstrated no significant difference (p≥0.765) among themselves (Fig 12C).

#### aPDT against triple-species biofilm

Table 2 shows the results of the treatments with free CUR, anionic and cationic formulations against the triple-species biofilm. Washed biofilms demonstrated reductions of 1 to 2 log_10_ compared with non-washed biofilms for each species.

**Table 2:** Mean values of log_10_(CFU/mL) and standard deviation of the triple-species biofilm for each group evaluated.

	Groups								
	C-L-		C-L+		N	C+L-		C+L+	
	not washed	washed	not washed	washed		not washed	washed	not washed	washed
**Free CUR**									
*S*. *mutans*	6.59 (0.27)^A^	5.18 (0.28)	6.60 (0.15)^A^	5.51 (0.10)	-	6.53 (0.21)^A^	5.26 (0.02)	5.54 (0.57)^B^	3.37 (0.62)
*C*. *albicans*	6.03 (0.51)^A^	5.09 (0.04)	5.84 (0.39)^A^	5.29 (0.10)	-	5.67 (0.96)^A^	5.17 (0.02)	3.62 (2.00)^B^	5.15 (0.07)
MRSA	7.53 (0.39)^A^	6.32 (0.13)	7.23 (0.44)^A^	6.56 (0.06)	-	7.30 (0.32)^A^	6.53 (0.03)	6.63 (0.45)^B^	5.25 (0.08)
**Anionic CUR-NP**									
*S*. *mutans*	7.57 (0.23)^A^	5.58 (0.23)	7.43 (0.42)^A^	5.47 (0.28)	7.39 (0.11)^A^	7.58 (0.25)^A^	5.56 (0.26)	7.48 (0.22)^A^	4.59 (0.25)
*C*. *albicans*	6.54 (0.19)^ABC^	5.42 (0.15)	6.64 (0.24)^ABC^	5.50 (0.23)	6.74 (0.12)^B^	6.55 (0.13)^ABC^	5.53 (0.11)	6.49 (0.17)^C^	5.40 (0.15)
MRSA	7.77 (0.18)^A^	6.63 (0.28)	7.67 (0.19)^A^	6.57 (0.14)	7.73 (0.16)^A^	7.64 (0.25)^A^	6.52 (0.26)	7.65 (0.14)^A^	6.26 (0.41)
**Cationic CUR-NP**									
*S*. *mutans*	7.72 (0.13)^A^	5.82 (0.25)	7.74 (0.16)^A^	5.73 (0.38)	3.23 (1.49)^B^	2.67 (1.81)^B^	4.19 (0.09)	3.28 (1.01)^B^	3.40 (0.23)
*C*. *albicans*	6.61 (0.31)^A^	5.34 (0.19)	6.47 (0.26)^A^	5.54 (0.16)	5.35 (0.53)^B^	4.29 (0.53)^C^	4.16 (0.40)	4.35 (0.53)^C^	3.59 (0.15)
MRSA	7.75 (0.27)^A^	6.44 (0.17)	7.81 (0.06)^A^	6.62 (0.21)	6.47 (0.26)^B^	5.23 (1.01)^C^	5.50 (0.28)	3.54 (1.51)^C^	5.24 (0.26)

-: not evaluated; different letters denote significant difference (p<0.05) for non-washed samples; washed samples: n = 6 for anionic and cationic CUR-NP, and n = 3 for free CUR in each group (no statistical inference was performed).

The results obtained for free CUR showed significant differences among the treatment groups for all microorganisms (p<0.001). The aPDT promoted a significant reduction in the viability of the three species evaluated in comparison to the control and the other treatments (p values were <0.001 for *S*. *mutans*, ≤0.007 for *C*. *albicans*, and ≤0.002 for MRSA). There was no significant difference among the other groups for *S*. *mutans* (p≥0.840), *C*. *albicans* (p≥0.668), and MRSA (p≥0.314). Compared with control (C-L-), the aPDT mediated by the free CUR promoted reductions of 1.05, 2.41, and 0.90 log_10_ for *S*. *mutans*, *C*. *albicans*, and MRSA, respectively. For washed samples submitted to aPDT, only bacteria showed reduction of viability compared with control (1.81 and 1.07 log_10_ for *S*. *mutans* and MRSA, respectively).

The evaluation of the triple-species biofilm after treatments with the anionic formulations demonstrated a significant difference only for *C*. *albicans* when the anionic groups N and C+L+ were compared (p = 0.036), while no significant difference among the other groups (p≥0.129) was observed. There was also no significant difference among the groups for *S*. *mutans* (p≥0.548) and MRSA (p≥0.579), since bacterial growth was observed after all treatments. After aPDT, washed samples showed reduction in viability only for bacteria (0.99 and 0.37 log_10_ for *S*. *mutans* and MRSA, respectively) compared with control.

When cationic formulations were compared, significant differences were observed for the three microorganisms, which showed similar growth behavior after the treatments. The cationic formulations, with or without CUR, in the presence or absence of light (NP, C+L-, and C+L+ groups), resulted in a significant reduction of log_10_(CFU/mL) values (p≤0.036) in comparison with the control group, with no significant difference between the C+L- and C+L+ groups (p≥0.088). There was no significant difference (p≥0.849) between the control group (C-L-) and the C-L+ groups in the log_10_(CFU/mL) values for the three microbial species. Compared with the control (C-L-), the highest reductions observed for *S*. *mutans*, *C*. *albicans*, and MRSA were 5.05 log_10_ (C+L- group), 2.32 log_10_ (C+L- group) and 4.21 log_10_ (C+L+ group), respectively. For washed samples, all species showed reduction in viability after aPDT (2.42, 1.75, and 1.2 log_10_ for *S*. *mutans*, *C*. *albicans*, and MRSA, respectively) and after being treated only with the cationic CUR-NP (1.63, 1.18, and 0.94 log_10_ for *S*. *mutans*, *C*. *albicans*, and MRSA, respectively) compared with control.

#### CSLM analysis

**Fig 13:**
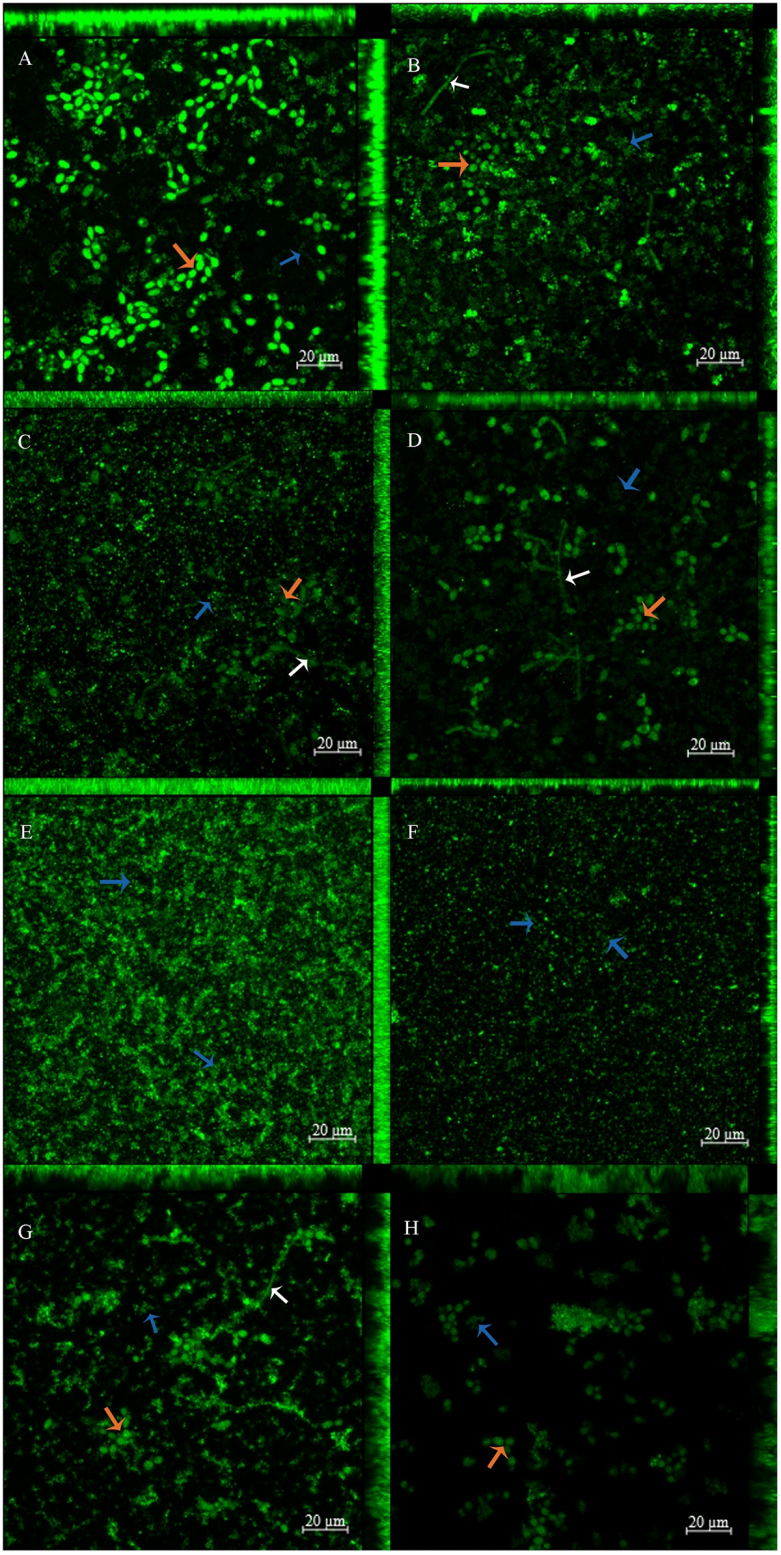
CLSM images showing uptake of free CUR (C and D), anionic CUR-NP (E and F), and cationic CUR-NP (G and H) by the triple-species biofilm. **Controls (A and B) were stained with SYTO-9**. **Samples were washed thrice with PBS (B, D, F, and H) or not washed at all (A, C, E, and G)**. Magnification: 20 μm. Blue arrows: coccus; red arrows: yeasts; white arrows: hyphaes.

The images showed brighter fluorescence for non-washed samples (Fig 13A, 13C, 13E and 13F) than those washed thrice with PBS (Fig 13B, 13D, 13F and 13G), which suggests that the washing step reduced the PS uptake. Control biofilms stained with SYTO-9 (Fig 13A and 13B) demonstrated the fluorescence of coccus, yeast, and hyphae, and a mean thickness of 14.62 μm and 12.9 μm for non-washed and washed samples, respectively. Biofilms incubated with free CUR (Fig 13C and 13D) showed a mean thickness of 6.24 μm (non-washed) and 7.02 μm (washed) and brighter fluorescence for coccus than for yeast, suggesting a better uptake of free CUR by the bacteria than by the yeast. For anionic CUR-NP no yeast was observed, only a high amount of coccus suggesting an uptake only by the bacteria, and a mean thickness of 7.46 μm and 7.31 μm for non-washed and washed samples, respectively. Finally, biofilms treated with cationic CUR-NP demonstrated fluorescence of coccus, yeast, and hyphae, suggesting that all microoganisms had taken up the cationic formulation, and a mean thickness of 9.75 μm (non-wahsed) and 13.47 μm (washed). Furthermore, washing the PSs did not reduce the biofilm thickness.

#### Cell viability assay

**Fig 14:**
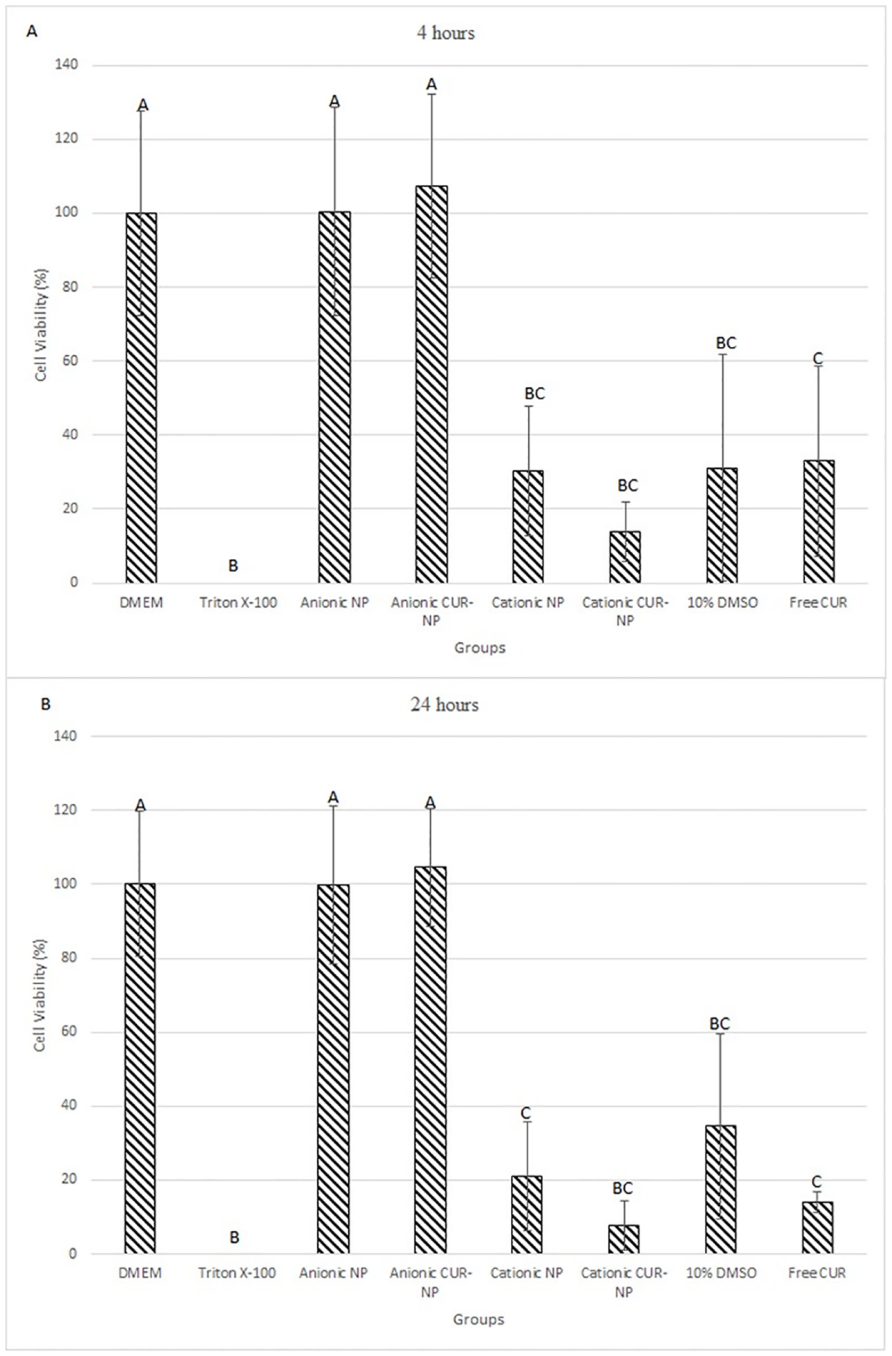
Cell viability (mean percentage of survival) after 4 hours (A) and 24 hours (B) of incubation with Alamar blue in all groups evaluated. Error bars: standard deviation. Different letters denote significant differences.

After 4 hours of incubation with Alamar blue (Fig 14A), cells treated with anionic formulations (NP and CUR-NP) showed no significant difference of viability (percentage of survival) compared between themselves and with the negative control group (p≥0.998). However, these groups showed significant difference compared to the others groups (p<0.001). On the other hand, cells incubated with cationic formulations (NP and CUR-NP), free CUR, and 10% DMSO did not show statistical differcence among themselves (p≥0.657). Additionaly, cells treated with cationic solutions (NP and CUR-NP) and 10% DMSO did not demonstrate any significant difference compared with the positive control group (Triton X-100).

Similar to the test performed at 4 hours, after 24 hours of incubation the negative control group and groups treated with anionic formulation (NP and CUR-NP) did not show significant difference among themselves (p≥0.999), and these groups showed significant difference (p≤0.001) compared with the other groups (positive control group, cationic NP, cationic CUR-NP, 10% DMSO, and free CUR). Additionaly, the positive control group also showed significant difference compared with those of cationic NP and free CUR (p≤0.049). However, the positive control group (Triton X-100) did not show any significant difference compared with groups treated with cationic CUR-NP and 10% DMSO (p≥0.061). Finally, for samples treated with cationic formulations (NP and CUR-NP), the organic solvent (10% DMSO) and free CUR, no significant difference was observed among them (p≥0.186) (Fig 14B).

## Discussion

Although previous studies have evaluated antimicrobial effects of CUR in nanocarriers, these studies were focused on the prevention of biofilm formation [19] or tested against planktonic cultures [18]. Only a few studies have used CUR in nanoformulations against planktonic oral species [20] or as PS in aPDT against non-oral biofilm [16]. Moreover, the susceptibility of different kinds of biofilms (mono-, dual-, and triple-species biofilms) to aPDT is not extensively explored. Thus, the present investigation evaluated the photodynamic effect of CUR-NP on planktonic cultures and mixed biofilms of microbial species found in the oral cavity. Due to good physico-chemical properties and capacity to encapsulate drugs, polymeric NP represents one of most successful nanocarrier [27]. In this research, we used PLA as polymer, since this compound is biodegradable, biocompatible, and its sub-products of degradation are non-toxic in superficial tissues. Moreover, PLA was approved for direct contact with biological fluids [28]. Furthermore, PLA could be degradated by microorganisms (fungi and bacteria) via esterease enzymatic action [29]. These features make PLA an attractive compound to use as a nanocarrier for antimicrobial purpose. In addition, the nanoprecipitation method used to synthesize CUR-NP in the present research is simple, reproductible, fast, economic, and easy to apply in industrial scale [30].

The results demonstrated that the encapsulation of CUR in NPs was performed successfully. When the formulations were characterized, their values of mean size, PDI, and zeta potential changed along time after synthesis, since at each time variations begin to appear. Although this was not the goal of this investigation, it could be suggested that these formulations may be stable for short periods after synthesis. The stability of these nanoformulations could be related to the fact that NPs with high zeta potential (in modulus) produces repulsion among the NPs avoiding NP aggregation [31]. Since the PDI values may range from 0 to 1, the PDI value obtained in this study may be considered homogeneous, although only values lower than 0.1 are associated to high homogeneity [32]. Moreover, the size of NP were smaller when they were evaluated by SEM compared with DLS, but this may a consequence of the shinkrage of the NP after the drying procedure for SEM [32].

The physicochemical properties of nanoparticles are related to the polymer and the method used to produce them. Polylactic co-glycolic acid (PLGA) was used in previous studies to synthesize nanoparticles containing CUR by different methods, such as oil/water emulsion-solvent evaporation [33], nanoprecipitation [23], and emulsion-difussion-evaporation [34]. The physicochemical properties obtained by these methods were, in general, similar to those obtained in the present study. The mean sizes, PDI, and zeta potential of the formulations synthesized by emulsion-solvent evaporation [33]/nanoprecipitation [23] methods were 176/76.2–560.4 nm, PDI 0.105/0.19–0.41 and -23.2/+0.06 and -0.06 mV, respectively [33,23]. Another study that used the emulsion-evaporation method obtained nanospheres with size of 264 nm [34]. Gosh et al. [35] prepared CUR-NPs with PLGA and observed by Atomic Force Microscopy nanoparticles with a diameter of 15 nm. PLGA was also used to synthesize NPs loaded with CUR by the single emulsion solvent evaporation technique, where particles with a size of 161.93 nm and a PDI of 0.042 were obtained [36]. PLA and PLGA are polymers approved by the FDA due to their biodegradability and biocompatibility. The difference between PLA and PLGA are the products produced after their metabolism in the body: while PLA breaks into monomers of lactic acid, PLGA produces monomers of lactic acid and glycolic acid [21].

The maximum absorbance of anionic and cationic CUR-NPs were 2.272 a.u. (425 nm) and 2.256 a.u. (426 nm), respectively, while the free CUR absorbance was 1.645 a.u. at 429 nm wavelength. These data are in agreement with the data found in other studies, which showed that CUR absorbs blue light at wavelength between 408–434 nm [11,18]. The anionic and cationic CUR-NPs showed a reduction in the absorbance band of 60.48% and 42.19%, respectively, after 5 min of irradiation, corresponding to 10.85 J/cm^2^, while the free CUR showed a decrease of 20.26% after the same light dose. The results observed with the CUR-NPs were similar to a previous investigation [16] that demonstrated a CUR degradation of 50% with a light dose of 9.4 J/cm^2^. However, Dovigo et al. [11], observed a degradation of 70% after a lower light dose of 0.4 J/cm^2^. These differences could be explained by the different concentrations of free CUR and CUR-NPs used in this study and in the another investigation [11].

In the present investigation, the EE percentage was 67% for the anionic CUR-NPs and 64.67% for the cationic CUR-NPs. Previous studies showed higher EE, varying from 78.95% to 97% [31,33,37,38]. The difference in the EE percentage could be explained by the centrifugation speed and time. In the present investigation, a centrifugation of 6,000 x*g* for 10 minutes was applied, while others studies have used higher velocities and longer times to determine the EE (15,000 rpm for 1 hour [31], 15,000 xg for 15 minutes [38]). This difference in the EE percentage could also be explained by the different methods employed in others studies, such as High Performance Liquid Chromatography (HPLC), which achieved EE values of 46.9, 77.07, 89, and 90% [33,36,39,40]. The chromatographic condition could be adjusted to the physicochemical characteristics of the nanocarrier without interfering in the structure of the polymeric nanoformulation, which is an advantage of the HPLC method over the UV-VIS method [41].

The present study also evaluated the CUR release from the NPs and the results showed a gradual release of 96% after 48 hours. Krausz et al. [17] obtained similar results, since a CUR release of 42.3% occured in the first 6 hours, achieving 81.5% after 24 hours. However, these high release values are different from those found in other studies, which demonstrated CUR releases of 40.5% and 75.7% after the first 24 hours and eight days [33], 21% and 56.9% after 24 hours and 9 days [36], and 20% and 80% after 24 hours and 360 hours [19]. When CUR was encapsulated in chitosan, alginate, and starch, release values of 92.8% after 96 hours, 51.4% after 24 hours, and 73.4% after 96 hours, respectively, were found [20]. These differences may be attributed to the polymers used for the encapsulation process and the methodology employed to evaluate the drug release (HPLC [36], dialysis membrane [31]).

Eradication of planktonic cultures of *S*. *mutans*, *C*. *albicans*, and MRSA was observed when free CUR was associated with blue light after 40 minutes of pre-irradiation and a light dose of 43.2 J/cm^2^. The light dose used in this research is similar to that used by Paschoal et al. [42], who used 42 J/cm^2^ for CUR at 0.75 μM, and they observed a reduction of 5.37 log_10_ in the planktonic culture of *S*. *mutans*. Another study showed that 15 mg/mL of CUR used as PS and a light dose of 5.7 J/cm^2^ yielded inactivation of *S*. *mutans* culture [43]. Regarding the photoinactivation of planktonic cultures of *C*. *albicans*, previous studies exhibited yeast eradication using CUR at 20 μM and a light dose of 5.28 J/cm^2^ [11,13]. In another study, aPDT mediated by CUR and blue light against MRSA planktonic cultures [44] was tested, and it showed a reduction of 9.8 log_10_, associated with free CUR at 20 μM and a light dose of 37.5 J/cm^2^.

In this study, 130 μM of CUR-NPs and free CUR were used for the antimicrobial evaluation of the planktonic cultures. A lower Minimum Inhibitory Concentration (MIC) was found in previous studies [10], corresponding to 23.5 μM against *C*. *albicans*. This could explain the reduction of 2.43 log_10_ of *C*. *albicans* and 0.75 log_10_ for *S*. *mutans* when treated only with free CUR (C+L- group), but this result was not observed for MRSA. MIC values of 0.128 mg/mL [45] and 125 μmol/L [46] have been reported against *S*. *mutans*, and values of 125–250 mg/mL [47] and 217 mg/mL [48] have been found for MRSA. MIC values of 6 mg/mL was also found for *S*. *aureus* and *S*. *mutans* and 1.5 mg/mL for *C*. *albicans*, but no microbicidal effect (absence of colonies growth) was observed for these species [31].

As expected, in the present investigation the planktonic cultures showed higher susceptibility to aPDT compared with the biofilms. Few studies showed photoinactivation of multispecies biofilm. Results obtained in this research using free CUR agree with previous studies which showed inhibition of dual-species biofilm composed of *S*. *mutans* and *Lactobacillus acidophilus* by aPDT mediated by CUR at 0.75, 1.5, and 3.0 g/L, resulting in reductions of 97.5, 95 and 99.9%, respectively, of microbial inactivation [43]. A higher concentration of PS was used in this research to inhibit the multispecies biofilm, similar to the study performed by Quishida et al. [49] who observed a significant reduction of a 48h-multispecies biofilm formed by *C*. *albicans*, *C*. *glabrata*, and *S*. *mutans* after aPDT mediated by CUR at 120 μM. In another study using a multispecies biofilm of *C*. *albicans*, *S*. *aureus*, and *S*. *mutans*, inhibitions of 1.00, 1.4,7 and 1.25 log_10_, respectively, were observed using 0.1 mg/mL of methylene blue as PS [50]. On the other hand, some studies applied aPDT against monospecies biofilm, and observed a reduction of 85% in the metabolism of *C*. *albicans* biofilm using CUR at a concentration of 40 μM and 20 minutes of pre-irradiation time with a light fluence of 18 J/cm^2^ [12].

The anionic CUR-NPs demonstrated an antimicrobial photodynamic effect against planktonic cultures, since a microbicidal effect was observed only when CUR-NPs were exposed to light, and no antimicrobial effect was verified for samples submitted to CUR-NPs without light, nor with NPs by themselves. However this photoinactivation was low, since there was no complete photoinactivation of the three microorganisms evaluated (*S*. *mutans*, *C*. *albicans*, and MRSA), as was observed with the free CUR. However, it's important to emphasize that free CUR at the same concentrarion of CUR-NP (260 μM) did not reduce the triple-species biofilm evaluated in this investigation (data not shown); so higher concentrations were evaluated and only 1,200 μM resulted in photoinactivation. When the anionic formulation was applied to the biofilms, only *S*. *mutans* was susceptible to aPDT in mono-species biofilm. Conversely, for the triple-species biofilm, no photodynamic inactivation was verified, since the three species were able to grow after aPDT similar to the control (C-L- group). This result could be explained by the surface charge (anionic) of the CUR-NPs, because some studies have shown that cationic PSs demonstrated an improved antimicrobial effect when compared with anionic ones [51,52]. Exopolysaccharides of the biofilm matrix have an anionic charge [53], which generates repulsion against anionic external agents. Therefore, the effect of anionic PSs is limited. Moreover, the slow release of CUR from the NPs demonstrated in this investigation may also explain the reduced antimicrobial photodynamic effect of anionic CUR-NP. In addition, washing the triple-species biofilm after incubation with free CUR and anionic CUR-NP abolished the photodynamic effect against *C*. *albicans*. This result corroborates with a previous investigation that demonstrated that washing CUR from planktonic suspension of *C*. *albicans* reduced the fungal photoinactivation [11].

Due to the limited effect of the anionic CUR-NP against biofilm, CTAB was incorporated in the formulation in order to produce cationic CUR-NP [22]. The results of the microbiological evaluation demonstrated that cationic nanoparticles, CUR-NPs, and NPs, had a microbicidal effect since they were able to eradicate the growth of the three microorganisms evaluated in the planktonic cultures. When these species were grown as biofilms, an antimicrobial effect against the three microorganisms was observed for cationic NPs with or without CUR, even in the absence of light, since reductions of colonies were observed. Even washing biofilms after incubation with cationic CUR-NP resulted in decreased viability for all species. Therefore, the reduction in the microbial viability observed for samples submitted to cationic formulations may be attributed to the their toxic effect to microbial cells, as also observed in the viability assay with mammalian cells (keratinocytes). The microbicidal effect could be explained by the CTAB incorporation in the formulation, since it was demonstrated that this compound causes lysis in the cell wall of *S*. *aureus*, *Enterococcus faecalis*, and *Escherichia coli* [54].

Several studies have demonstrated antimicrobial activity of CUR-NP, but only against planktonic cultures. The minimum inhibitory concentration (MIC) of 100 μg/mL of the CUR-NPs synthesized by the wet milling technique in planktonic cultures of *S*. *aureus* compared to CUR in DMSO (150 mg/mL) was observed in a previous study [55]. Another investigation demonstrated a MIC of 30 mg/mL against planktonic MRSA cultures using up-conversion nanoparticles conjugated with CUR after infrared light irradiation for 30 minutes [56]. Nanoparticles containing CUR produced a reduction of 97% in the viability of the planktonic MRSA cultures after incubation of 4 hours [17]. Hazzah et al. [31] synthesized solid lipid CUR-NPs and they observed lower MIC values against *S*. *aureus*, *S*. *mutans*, and *C*. *albicans* compared with raw CUR. CUR nanospheres at 1 mg/mL inhibited *E*. *coli*, *S*. *aureus*, and *C*. *albicans* after incubation of 24 hours [57]. CUR loaded in chitosan, alginate and starch showed MIC values of 0.114, 0.204, and 0.204 mg/mL, lower than free CUR (0.438 mg/mL), to inhibit *S*. *mutans* growth [20]. Inhibition zones of 17 mm for MRSA and *S*. *aureus*, 18 mm for *E*. *coli* and *Klebsiella pneumonia*, both resistant and not resistant to extended-spectrum β-lactamase, was observed on agar culture using electro-spun CUR loaded into poly-(2-hydroxyethyl methacrylate) nanofiber incubated overnight on suspensions of these microorganisms [58].

Few studies have evaluated aPDT mediated by CUR in nanocarriers. Baltazar et al. [18] found complete inactivation of the planktonic culture of *T*. *rubrum* treated with a nanoparticle containing CUR at 10 μg/mL for 10 minutes of pre-irradiation time and blue light at 10 J/cm^2^. Fungal growth was inhibited by 7 days after treatment. An aquous solution of CUR in polyvinylpyrrolidone (PVP-C) at 5 μM incubated for 5 minutes with planktonic cultures of *S*. *aureus* followed by a light fluence of 33.8 J/cm^2^ resulted in bacterial photoinactivation of more than 6 log_10_. Increasing the PVP-C concentration to 50 μM and the incubation period to 25 minutes prior illumination resulted in the complete eradication of the Gram(+) bacterium [59]. When aPDT was used for food decontamination, PVP-C at 50 μM and at 100 μM immediately associated to the same light fluence reduced *S*. *aureus* in 2.6 log_10_ and 2.5 log_10_ from cucumber and pepper [60]. In an ex vivo porcine model, the same parameters of aPDT mediated by PVP-C resulted in a higher reduction (1.7 log_10_ and 1.3 log_10_) of *S*. *aureus* than those observed for *E*. *coli* (0.0 log_10_ and 0.3 log_10_). When a cationic derivative of CUR at 50 μM and 100 μM was associated to light at 33.8 J/cm^2^, photoinactivation of the Gram(-) species was improved, since reductions of 2.2 log_10_ and 1.8 log_10_ for *S*. *aureus* and 3.2 log_10_ and 3.3 log_10_ for *E*. *coli* were observed [61].

The results of the present investigation against biofilms agree with other studies, in which erradication of biofilm was not observed. Hegge et al. [16], observed less than 1 log_10_ reduction of *S*. *epidermidis* biofilm after aPDT mediated by CUR solubilized in polyethylene glycol 400, pluronic F127, and hydroxypropyl-γ-cyclodextrin. CUR in micellar NP combined with silver NP at 30 μg/mL CUR and 3.75 μg/mL Ag supressed the biofilm formation of *S*. *aureus* and *P*. *aeruginosa*. However, a higher concentration (400 μg/mL of CUR-NP and 50 μg/mL of AgNP) was necessary to reduce by 70% the biomass of pre-formed biofilms [19]. Inhibition of biofilm formation from 89.48% to 99.38% was observed when *S*. *mutans* was incubated with CUR loaded in polysaccharide NP [20]. In the present investigation, the susceptibility of each species to aPDT varied, and the reduction rates (near to 1–3 log_10_) were similar in mono-, dual-, and triple-species biofilms. Conversely, Pereira et al. verified that singlespecies biofilm of *S*. *mutans*, *S*. *aureus* and *C*. *albicans* showed a higher susceptibility to aPDT mediated by methylene blue and laser compared with dual- and triple-species biofilms [50]. The difference between the outcomes of these studies may be atributted to the different PS, light source, and aPDT parameters employed for biofilm inactivation.

In the present investigation, anionic CUR-NP showed the lowest antimicrobial photodynamic effect, while cationic CUR-NP was cytotoxic. However, free CUR needed a concentration 4.62 times higher than CUR-NPs to promote a significant reduction in mixed-species biofilms. Since the maximum CUR concentration able to be encapsulated in NP was 260 μM, we couldn't conclude that CUR-NP was more effective than free CUR. Regarding the biological relevance, several investigations reported that, according to the American Society for Microbiology and CLSI standards, reduction of 3 log_10_ are considered to be biologically relevant [62,63,64,65]. Thus, although the results of this investigation showed significant reductions of log_10_(CFU/ml) values, reductions lower than 3 log_10_, such as those observed for some biofilms, may not be considered biologically relevant. Future studies should be conducted in order to improve the formulation of CUR and the efficacy of aPDT.

CUR is auto-fluorescent and emits a green fluorescence [37]. In this research, confocal microscopy showed that washing the PS from the biofilm reduced the fluorescence, although its intensity was not measured. This result agrees with that of the viability assay [log_10_(CFU/mL)] with washed biofilms, which demonstrated lower values compared with non-washed biofilms. Therefore, washing the PS reduced the photoinactivation of the biofilm, as demonstrated in previous studies that report that CUR in the bulk solution increases the photoinactivation of bacteria [66] and mono-species biofilm of *C*. *albicans* [11]. While cationic CUR-NP showed uptake by all cell morphologies found in the triple-species biofilm evaluated, free CUR demonstrated better uptake by bacteria, but anionic CUR-NP was not taken up by yeast, which justify the low photoinactivation of planktonic *C*. *albicans* with anionic CUR-NP (only 0.78 log_10_). This result was similar to that obtained in another study performed with free CUR, in which CUR was taken up by *S*. *aureus* [58]. The mechanism of incorporating the CUR-NPs into the bacteria is the fixation of the PS onto the bacterial wall, followed by breaking the peptidoglycan layer and finally the penetration of the PS to within the microorganism [55,67,68]. Andrade et al. [13] observed CUR uptake in *C*. *albicans* after a five- and a twenty-minute incubation using confocal laser scanning microscopy. In the present investigation, the biofilm labeled with SYTO-9 (control group) was thicker than those treated with free CUR and anionic CUR-NP, suggesting that these PSs were not able to penetrate deeply into the biofilm. This result agrees with Andrade et al. [13], who reported that CUR does not photosensitise the deepest cells inside the biofilm, but only the cells located in the outermost layers, which may explain the incomplete inactivation of the biofilm in this study. On the other hand, cationic CUR-NP showed thickness values more similar to the control group, which suggests that the atractive force between the positive charge of the formulation and the negative charge of the biofilm increases the PS penetration into the biofilm. However, it is important to emphasize that the thickness measured in the z-stack produced by the confocal microscope is related to the fluorescence of the labeled biofilm. This being the case, the deepest layers of cells may not have been labeled even by the SYTO-9, since it was used on pre-formed biofilms. Ideally, the biofilm should grow in the presence of the fluorophore to label the whole biofilm and for its thickness to be measured accurately.

In the present research, the anionic formulations (NP and CUR-NP) showed a similar cell viability percentage compared to the negative control group cultured with DMEM, which demonstrates that the anionic CUR-NP developed in this investigation is not cytotoxic. It confirms that PLA is a biocompatible polymer having low toxicity and is an attractive compound to encapsulate drugs. Similar to keratinocytes treated with Triton X-100, cationic CUR-NP and 10% DMSO (free CUR vehicle) showed cellular toxicity. Although samples treated with cationic NP after 24 hours and with free CUR in both periods showed significant difference compared with the positive control group (Triton X-100), they demonstrated cell viabilities under 40%, which is considered cytotoxic [69]. As observed in the microbiological assays, the cytotoxicity observed for cationic formulations (NP and CUR-NP) may be atributed to CTAB. Moreover, the cytotoxic effect of free CUR may be explained by the high concentration of DMSO (10%) used in the present investigation, since the same concentration used in antimicrobial assays was evaluated in the cytotoxic assay. However, the concentration of DMSO recommended to test cell viability is lower than 1% [70]. Therefore, the encapsulation of CUR with NP overcomes the cytotoxic effect of organic solvents.

In conclusion, encapsulation of CUR in polymeric nanoparticles was performed successfully using PLA as polymer, and the properties of the CUR-NPs were similar to those of the free CUR. The anionic CUR-NPs showed reduced photoinactivation of biofilms and no cytotoxicity, while the cationic CUR-NPs and NPs showed antimicrobial activity even without light but they were cytotoxic. However, only mature biofilms were evaluated in this study. It was demonstrated that micro-organisms growing in the presence of CUR-NP resulted in inhibition of biofilm formation [19,20]. Nonetheless, the ability of CUR to inhibit multispecies biofilm formation was not assessed in this investigation. In addition, further studies developing cationic systems of faster release of drugs should be investigated for aPDT.

## References

[1] Lemos JA, Quivey RG Jr, Koo H, Abranches J. *Streptococcus mutans*: a new Gram-positive paradigm?. Microbiology. 2013;159: 436–445. doi: 10.1099/mic.0.066134-0 23393147 10.1099/mic.0.066134-0 PMC4083656

[2] Sardi JC, Scorzoni L, Bernardi T, Fusco-Almeida AM, Mendes Giannini MJ. *Candida* species: current epidemiology, pathogenicity, biofilm formation, natural antifungal products and new therapeutic options. J Med Microbiol. 2013;62: 10–24. doi: 10.1099/jmm.0.045054-0 23180477 10.1099/jmm.0.045054-0

[3] Cassone A, Cauda R. *Candida* and candidiasis in HIV-infected patients: where commensalism, opportunistic behavior and frank pathogenicity lose their borders. AIDS. 2012;26: 1457–1472. doi: 10.1097/QAD.0b013e3283536ba8 22472853 10.1097/QAD.0b013e3283536ba8

[4] Oud L. Secular trends in utilization of critical care services among candidemia-associated hospitalizations: a population-based cohort study. J Clin Med Res. 2016;8: 40–43. doi: 10.14740/jocmr2387w 26668681 10.14740/jocmr2387w PMC4676344

[5] Russotto V, Cortegiani A, Graziano G, Saporito L, Raineri SM, Mammina C, et al Bloodstream infections in intensive care unit patients: distribution and antibiotic resistance of bacteria. Infect Drug Resist. 2015;10: 287–296. 10.2147/IDR.S48810 PMC4536838 26300651

[6] Zago CE, Silva S, Sanitá PV, Barbugli PA, Dias CM, Lordello VB, et al Dynamics of biofilm formation and the interaction between *Candida albicans* and methicillin-susceptible (MSSA) and -resistant *Staphylococcus aureus* (MRSA). PloS One. 2015;10: e0123206 doi: 10.1371/journal.pone.0123206 25875834 10.1371/journal.pone.0123206 PMC4395328

[7] Falsetta ML, Klein MI, Colonne PM, Scott-Anne K, Gregoire S, Pai CH, et al Symbiotic relationship between *Streptococcus mutans* and *Candida albicans* synergizes virulence of plaque biofilms in vivo. Infect Immun. 2014;82: 1968–1981. doi: 10.1128/IAI.00087-14 24566629 10.1128/IAI.00087-14 PMC3993459

[8] Soukos NS, Goodson JM. Photodynamic therapy in the control of oral biofilms. Periodontol 2000. 2011;55: 143–166. doi: 10.1111/j.1600-0757.2010.00346.x 21134233 10.1111/j.1600-0757.2010.00346.x

[9] Hamblim MR Antimicrobial photodynamic inactivation: a bright new technique to kill resistant microbes. Curr Opin Microbiol. 2016;33: 67–73. doi: 10.1016/j.mib.2016.06.008 27421070 10.1016/j.mib.2016.06.008 PMC5069151

[10] Martins CV, da Silva DL, Neres AT, Magalhães TF, Watanabe GA, Modolo LV, et al Curcumin as a promising antifungal of clinical interest. J Antimicrob Chemother. 2009;63: 337–339. doi: 10.1093/jac/dkn488 19038979 10.1093/jac/dkn488

[11] Dovigo LN, Pavarina AC, Ribeiro AP, Brunetti IL, Costa CA, Jacomassi DP, et al Investigation of the photodynamic effects of curcumin against *Candida albicans*. Photochem Photobiol. 2011;87: 895–903. doi: 10.1111/j.1751-1097.2011.00937.x 21517888 10.1111/j.1751-1097.2011.00937.x

[12] Dovigo LN, Pavarina AC, Carmello JC, Machado AL, Brunetti IL, Bagnato VS. Susceptibility of clinical isolates of *Candida* to photodynamic effects of curcumin. Lasers Surg Med. 2011;43: 927–934. doi: 10.1002/lsm.21110 22006736 10.1002/lsm.21110

[13] Andrade MC, Ribeiro AP, Dovigo LN, Brunetti IL, Giampaolo ET, Bagnato VS, et al Effect of different pre-irradiation times on curcumin-mediated photodynamic therapy against planktonic cultures and biofilms of *Candida* spp. Arch Oral Biol. 2013;58: 200–210. doi: 10.1016/j.archoralbio.2012.10.011 23153629 10.1016/j.archoralbio.2012.10.011

[14] Vrignaud S, Benoit JP, Saulnier P. Strategies for the nanoencapsulation of hydrophilic molecules in polymer-based nanoparticles. Biomaterials. 2011;32: 8593–8604. doi: 10.1016/j.biomaterials.2011.07.057 21831421 10.1016/j.biomaterials.2011.07.057

[15] Mohamed F, van der Walle CF. Engineering biodegradable polyester particles with specific drug targeting and drug release properties. J Pharm Sci. 2008;97: 71–87. doi: 10.1002/jps.21082 17722085 10.1002/jps.21082

[16] Hegge AB, Bruzell E, Kristensen S, Tønnesen HH. Photoinactivation of *Staphylococcus epidermidis* biofilms and suspensions by the hydrophobic photosensitizer curcumin—effect of selected nanocarrier: studies on curcumin and curcuminoides XLVII. Eur J Pharm Sci. 2012;47: 65–74. doi: 10.1016/j.ejps.2012.05.002 22609527 10.1016/j.ejps.2012.05.002

[17] Krausz AE, Adler BL, Cabral V, Navati M, Doerner J, Charafeddine RA, et al Curcumin-encapsulated nanoparticles as innovative antimicrobial and wound healing agent. Nanomedicine. 2015;11: 195–206. doi: 10.1016/j.nano.2014.09.004 25240595 10.1016/j.nano.2014.09.004 PMC4461434

[18] Baltazar LM, Krausz AE, Souza AC, Adler BL, Landriscina A, Musaev T, et al *Trichophyton rubrum* is inhibited by free and nanoparticle encapsulated curcumin by induction of nitrosative stress after photodynamic activation. PLoS ONE. 2015;10: e0120179 doi: 10.1371/journal.pone.0120179 25803281 10.1371/journal.pone.0120179 PMC4372525

[19] Loo CY, Rohanizadeh R, Young PM, Traini D, Cavaliere R, Whitchurch CB, et al Combination of silver nanoparticles and curcumin nanoparticles for enhanced anti-biofilm activities. J Agric Food Chem. 2016;64: 2513–2522. doi: 10.1021/acs.jafc.5b04559 26595817 10.1021/acs.jafc.5b04559

[20] Maghsoudi A, Yazdian F, Shahmoradi S, Ghaderi L, Hemati M, Amoabediny G. Curcumin-loaded polysaccharide nanoparticles: optimization and anticariogenic activity against *Streptococcus mutans*. Mater Sci Eng C Mater Biol Appl. 2017;75: 1259–1267. doi: 10.1016/j.msec.2017.03.032 28415415 10.1016/j.msec.2017.03.032

[21] Kumari A, Yadav SK, Yadav SC. Biodegradable polymeric nanoparticles based drug delivery systems. Colloids Surf B Biointerfaces. 2010;75: 1–18. doi: 10.1016/j.colsurfb.2009.09.001 19782542 10.1016/j.colsurfb.2009.09.001

[22] Zou W, Liu C, Chen Z, Zhang N. Preparation and characterization of cationic PLA-PEG nanoparticles for delivery of plasmid DNA. Nanoscale Res Lett. 2009;4: 982–992. doi: 10.1007/s11671-009-9345-3 20596550 10.1007/s11671-009-9345-3 PMC2893611

[23] Yallapu MM, Gupta BK, Jaggi M, Chauhan SC. Fabrication of curcumin encapsulated PLGA nanoparticles for improved therapeutic effects in metastatic cancer cells. J Colloid Interface Sci. 2010;351: 19–29. doi: 10.1016/j.jcis.2010.05.022 20627257 10.1016/j.jcis.2010.05.022

[24] Castilho RM, Squarize CH, Leelahavanichk L K, Zheng Y, Bugge T, Gutkind JS. Rac1 is required for epithelial stem cell function during dermal and oral mucosal wound healing but not for tissue homeostasis in mice. PLoS One. 2010;5: e10503 doi: 10.1371/journal.pone.0010503 20463891 10.1371/journal.pone.0010503 PMC2865533

[25] de Carvalho Dias K, Barbugli PA, de Patto F, Lordello VB, de Aquino Penteado L, Medeiros AI, Vergani CE. Soluble factors from biofilm of *Candida albicans* and *Staphylococcus aureus* promote cell death and inflammatory response. BMC Microbiol. 2017;17: 146 doi: 10.1186/s12866-017-1031-5 28666415 10.1186/s12866-017-1031-5 PMC5493077

[26] O'Brien J, Wilson I, Orton T, Pognan F. Investigation of the Alamar Blue (resazurin) fluorescent dye for the assessment of mammalian cell cytotoxicity. Eur J Biochem. 2000;267: 5421–5426. 10951200 10.1046/j.1432-1327.2000.01606.x

[27] Couvreur P, Barratt G, Fattal E, Legrand P, Vauthier C. Nanocapsule technology: a review. Crit Rev Ther Drug Carrier Syst. 2002;19: 99–134. 12197610 10.1615/critrevtherdrugcarriersyst.v19.i2.10

[28] Farah S, Anderson DG, Langer R. Physical and mechanical properties of PLA, and their functions in widespread applications—A comprehensive review. Adv Drug Deliv Rev. 2016;107: 367–392. doi: 10.1016/j.addr.2016.06.012 27356150 10.1016/j.addr.2016.06.012

[29] Shah AA, Kato S, Shintani N, Kamini NR, Nakajima-Kambe T. Microbial degradation of aliphatic and aliphatic-aromatic co-polyesters. Appl Microbiol Biotechnol. 2014;98: 3437–3447. doi: 10.1007/s00253-014-5558-1 24522729 10.1007/s00253-014-5558-1

[30] Vauthier C, Bouchemal K. Methods for the preparation and manufacture of polymeric nanoparticles. Pharm Res. 2009;26: 1025–1058. doi: 10.1007/s11095-008-9800-3 19107579 10.1007/s11095-008-9800-3

[31] Hazzah HA, Farid RM, Nasra MM, Hazzah WA, El-Massik MA, Abdallah OY. Gelucire-based nanoparticles for curcumin targeting to oral mucosa: preparation, characterization, and antimicrobial activity assessment. J Pharm Sci. 2015;104: 3913–3924. doi: 10.1002/jps.24590 26202796 10.1002/jps.24590

[32] Gaumet M, Vargas A, Gurny R, Delie F. Nanoparticles for drug delivery: the need for precision in reporting particle size parameters. Eur J Pharm Biopharm. 2008;69: 1–9. doi: 10.1016/j.ejpb.2007.08.001 17826969 10.1016/j.ejpb.2007.08.001

[33] Chereddy KK, Coco R, Memvanga PB, Ucakar B, des Rieux A, Vandermeulen G, et al Combined effect of PLGA and curcumin on wound healing activity. J Control Release. 2013;171: 208–215. doi: 10.1016/j.jconrel.2013.07.015 23891622 10.1016/j.jconrel.2013.07.015

[34] Shaikh J, Ankola D, Beniwal V, Singh D, Kumar MN. Nanoparticle encapsulation improves oral bioavailability of curcumin by at least 9-fold when compared to curcumin administered with piperine as absorption enhancer. Eur J Pharm Sci. 2009;37: 223–230. doi: 10.1016/j.ejps.2009.02.019 19491009 10.1016/j.ejps.2009.02.019

[35] Ghosh D, Choudhury ST, Ghosh S, Mandal AK, Sarkar S, Ghosh A, et al Nanocapsulated curcumin: oral chemopreventive formulation against diethylnitrosamine induced hepatocellular carcinoma in rat. Chem Biol Interact. 2012;195: 206–214. doi: 10.1016/j.cbi.2011.12.004 22197969 10.1016/j.cbi.2011.12.004

[36] Khalil NM, do Nascimento TC, Casa DM, Dalmolin LF, de Mattos AC, Hoss I, et al Pharmacokinetics of curcumin-loaded PLGA and PLGA-PEG blend nanoparticles after oral administration in rats. Colloids Surf B Biointerfaces. 2013;101: 353–360. doi: 10.1016/j.colsurfb.2012.06.024 23010041 10.1016/j.colsurfb.2012.06.024

[37] Udompornmongkol P, Chiang BH. Curcumin-loaded polymeric nanoparticles for enhanced anti-colorectal cancer applications. J Biomater Appl. 2015;30: 537–546. doi: 10.1177/0885328215594479 26170212 10.1177/0885328215594479

[38] Mukerjee A, Vishwanatha JK. Formulation, characterization and evaluation of curcumin-loaded PLGA nanospheres for cancer therapy. Anticancer Res. 2009;29: 3867–3875. 19846921

[39] Tsai YM, Chien CF, Lin LC, Tsai TH. Curcumin and its nano-formulation: the kinetics of tissue distribution and blood–brain barrier penetration. Int J Pharm. 2011;416: 331–338. doi: 10.1016/j.ijpharm.2011.06.030 21729743 10.1016/j.ijpharm.2011.06.030

[40] Mohanty C, Sahoo SK. The in vitro stability and in vivo pharmacokinetics of curcumin prepared as an aqueous nanoparticulate formulation. Biomaterials. 2010;31: 6597–6611. doi: 10.1016/j.biomaterials.2010.04.062 20553984 10.1016/j.biomaterials.2010.04.062

[41] Sartori T, Seigi Murakami F, Pinheiro Cruz A, Machado de Campos A. Development and validation of a fast RP-HPLC method for determination of methotrexate entrapment efficiency in polymeric nanocapsules. J Chromatogr Sci. 2008;46: 505–509. 18647471 10.1093/chromsci/46.6.505

[42] Paschoal MA, Tonon CC, Spolidório DMP, Bagnato VS, Giusti JSM, Santos-Pinto L. Photodynamic potential of curcumin and blue LED against *Streptococcus mutans* in a planktonic culture. Photodiagnosis Photodyn Ther. 2013;10: 313–319. doi: 10.1016/j.pdpdt.2013.02.002 23993858 10.1016/j.pdpdt.2013.02.002

[43] Araújo NC, Fontana CR, Bagnato VS, Gerbi ME. Photodynamic effects of curcumin against cariogenic pathogens. Photomed Laser Surg. 2012;30: 393–399. doi: 10.1089/pho.2011.3195 22693952 10.1089/pho.2011.3195

[44] Ribeiro AP, Pavarina AC, Dovigo LN, Brunetti IL, Bagnato VS, Vergani CE, et al Phototoxic effect of curcumin on methicillin-resistant *Staphylococcus aureus* and L929 fibroblasts. Lasers Med Sci. 2013;28: 391–398. doi: 10.1007/s10103-012-1064-9 22358772 10.1007/s10103-012-1064-9

[45] Song J, Choi B, Jin EJ, Yoon Y, Choi KH. Curcumin suppresses *Streptococcus mutans* adherence to human tooth surfaces and extracellular matrix proteins. Eur J Clin Microbiol Infect Dis. 2012;31: 1347–1352. doi: 10.1007/s10096-011-1448-y 22009290 10.1007/s10096-011-1448-y

[46] Hu P, Huang P, Chen MW. Curcumin reduces *Streptococcus mutans* biofilm formation by inhibiting sortase A activity. Arch Oral Biol. 2013;58: 1343–1348. doi: 10.1016/j.archoralbio.2013.05.004 23778072 10.1016/j.archoralbio.2013.05.004

[47] Moghadamtousi SZ, Kadir HA. Hassandarvish P, Tajik H, Abubakar S, Zandi K. A review on antibacterial, antiviral, and antifungal activity of curcumin. BioMed Res Int. 2014;2014: 186864 doi: 10.1155/2014/186864 24877064 10.1155/2014/186864 PMC4022204

[48] Gunes H, Gulen D, Mutlu R, Gumus A, Tas T, Topkaya AE. Antibacterial effects of curcumin: an in vitro minimum inhibitory concentration study. Toxicol Ind Health. 2016;32: 246–250. doi: 10.1177/0748233713498458 24097361 10.1177/0748233713498458

[49] Quishida CC, De Oliveira Mima EG, Jorge JH, Vergani CE, Bagnato VS, Pavarina AC. Photodynamic inactivation of a multispecies biofilm using curcumin and LED light. Lasers Med Sci. 2016;31: 997–1009. doi: 10.1007/s10103-016-1942-7 27126412 10.1007/s10103-016-1942-7

[50] Pereira CA, Romeiro RL, Costa AC, Machado AK, Junqueira JC, Jorge AO. Susceptibility of *Candida albicans*, *Staphylococcus aureus*, and *Streptococcus mutans* biofilms to photodynamic inactivation: an in vitro study. Lasers Med Sci. 2011;26: 341–348. doi: 10.1007/s10103-010-0852-3 21069408 10.1007/s10103-010-0852-3

[51] Junqueira JC, Jorge AO, Barbosa JO, Rossoni RD, Vilela SF, Costa AC, et al Photodynamic inactivation of biofilms formed by *Candida* spp., *Trichosporon mucoides*, and *Kodamaea ohmeri* by cationic nanoemulsion of zinc 2,9,16,23-tetrakis(phenylthio)-29H, 31H-phthalocyanine (ZnPc). Lasers Med Sci. 2012;27: 1205–1212. doi: 10.1007/s10103-012-1050-2 22278349 10.1007/s10103-012-1050-2

[52] Ribeiro AP, Andrade MC, Bagnato VS, Vergani CE, Primo FL, Tedesco AC, et al Antimicrobial photodynamic therapy against pathogenic bacterial suspensions and biofilms using chloro-aluminum phthalocyanine encapsulated in nanoemulsions. Lasers Med Sci. 2015;30: 549–559. doi: 10.1007/s10103-013-1354-x 23748800 10.1007/s10103-013-1354-x

[53] Al-Fattani MA, Douglas LJ. Biofilm matrix of *Candida albicans* and *Candida tropicalis*: chemical composition and role in drug resistance. J Med Microbiol. 2006;55: 999–1008. doi: 10.1099/jmm.0.46569-0 16849719 10.1099/jmm.0.46569-0

[54] Salton MR. The adsorption of cetyltrimethylammonium bromide by bacteria, its action in releasing cellular constituents and its bactericidal effects. J Gen Microbiol. 1951;5: 391–404. doi: 10.1099/00221287-5-2-391 14832428 10.1099/00221287-5-2-391

[55] Mun SH, Kim SB, Kong R, Choi JG, Kim YC, Shin DW, et al Curcumin reverse methicillin resistance in *Staphylococcus aureus*. Molecules. 2014;19: 18283–18295. doi: 10.3390/molecules191118283 25389660 10.3390/molecules191118283 PMC6271166

[56] Ye Y, Li Y, Fang F. Upconversion nanoparticles conjugated with curcumin as a photosensitizer to inhibit methicillin-resistant *Staphylococcus aureus* in lung under near infrared light. Int J Nanomedicine. 2014;9: 5157–5165. doi: 10.2147/IJN.S71365 25395852 10.2147/IJN.S71365 PMC4226522

[57] Arunraj TR, Sanoj Rejinold N, Mangalathilham S, Saroj S, Biswas R, Jayakumar R. Synthesis, characterization and biological activities of curcumin nanospheres. J Biomed Nanotech. 2014;10: 238–250. 10.1166/jbn.2014.1786 24738332

[58] Ramalingam N, Natarajan TS, Rajiv S. Preparation and characterization of electrospun curcumin loaded poly(2-hydroxyethyl methacrylate) nanofiber-a biomaterial for multidrug resistant organisms. J Biomed Mater Res A. 2015;103: 16–24. doi: 10.1002/jbm.a.35138 24578218 10.1002/jbm.a.35138

[59] Winter S, Tortik N, Kubin A, Krammer B, Plaetzer K. Back to the roots: photodynamic inactivation of bacteria based on water-soluble curcumin bound to polyvinylpyrrolidone as a photosensitizer. Photochem Photobiol Sci. 2013;12: 1795–1802. doi: 10.1039/c3pp50095k 23828307 10.1039/c3pp50095k

[60] Tortik N, Spaeth A, Plaetzer K. Photodynamic decontamination of foodstuff from *Staphylococcus aureus* based on novel formulations o curcumin. Photochem Photobiol Sci. 2014;13: 1402–1409. doi: 10.1039/c4pp00123k 24957403 10.1039/c4pp00123k

[61] Tortik N, Steinbacher P, Maisch T, Spaeth A, Plaetzer K. A comparative study on the antibacterial photodynamic efficiency of a curcumin derivative and a formulation on a porcine skin model. Photochem Photobiol Sci. 2016;15: 187–195. doi: 10.1039/c5pp00393h 26751838 10.1039/c5pp00393h

[62] Nakonieczna J. Comment on "Effectiveness of antimicrobial photodynamic therapy (AmPDT) on *Staphylococcus aureus* using phenothiazine compound with red laser". Lasers Med Sci. 2017;32: 1667–1668. 10.1007/s10103-016-2107-4 27832391

[63] Cieplik F, Tabenski L, Buchalla W, Maisch T. Antimicrobial photodynamic therapy for inactivation of biofilms formed by oral key pathogens. Front Microbiol. 2014;5: 405 doi: 10.3389/fmicb.2014.00405 25161649 10.3389/fmicb.2014.00405 PMC4130309

[64] Taraszkiewicz A, Grinholc M, Bielawski KP, Kawiak A, Nakonieczna J. Imidazoacridinone derivatives as efficient sensitizers in photoantimicrobial chemotherapy. Appl Environ Microbiol. 2013;79: 3692–3702. doi: 10.1128/AEM.00748-13 23563951 10.1128/AEM.00748-13 PMC3675932

[65] Kiesslich T, Gollmer A, Maisch T, Berneburg M, Plaetzer K. A comprehensive tutorial on in vitro characterization of new photosensitizers for photodynamic antitumor therapy and photodynamic inactivation of microorganisms. Biomed Res Int. 2013;2013: 840417 doi: 10.1155/2013/840417 23762860 10.1155/2013/840417 PMC3671303

[66] Dahl TA, McGowan WM, Shand MA, Srinivasan VS. Photokilling of bacteria by the natural dye curcumin. Arch Microbiol. 1989;151: 183–185. 2655550 10.1007/BF00414437

[67] Tyagi P, Singh M, Kumari H, Kumari A, Mukhopadhyay K. Bactericidal activity of curcumin I is associated with damaging of bacterial membrane. PLoS ONE. 2015;10: e0121313 doi: 10.1371/journal.pone.0121313 25811596 10.1371/journal.pone.0121313 PMC4374920

[68] Basniwal RK, Buttar HS, Jain VK, Jain N. Curcumin nanoparticles: preparation, characterization, and antimicrobial study. J Agric Food Chem. 2011;59: 2056–2061. doi: 10.1021/jf104402t 21322563 10.1021/jf104402t

[69] Hamid R, Rotshteyn Y, Rabadi L, Parikh R, Bullock P. Comparison of alamar blue and MTT assays for high through-put screening. Toxicol in Vitro. 2004;18: 703–710. doi: 10.1016/j.tiv.2004.03.012 15251189 10.1016/j.tiv.2004.03.012

[70] Trivedi AB, Kitabatake N, Doi E. Toxicity of dimethyl sulfoxide as a solvent in bioassay system with HeLa cells evaluated colorimetrically with 3-(4,5-dimethyl thiazol-2-yl)-2,5-diphenyl-tetrazolium bromide. Agric Biol Chem. 1990;54: 2961–2966. 1368650

